# Reconstructed Ancestral Sequences Improve Pathogen Identification Using Resequencing DNA Microarrays

**DOI:** 10.1371/journal.pone.0015243

**Published:** 2010-12-20

**Authors:** Nicolas Berthet, Alexis Deletoile, Virginie Passet, Giulia C. Kennedy, Jean-Claude Manuguerra, Stewart T. Cole, Sylvain Brisse

**Affiliations:** 1 Institut Pasteur, Genotyping of Pathogens and Public Health, Paris, France; 2 Institut Pasteur, Epidemiology and Pathophysiology Oncogenic Virus Unit, CNRS URA3015, Paris, France; 3 Affymetrix, Santa Clara, California, United States of America; 4 Institut Pasteur, Laboratory for Urgent Responses to Biological Threats, Paris, France; 5 Global Health Institute, Laboratory of Microbial Pathogenesis, Ecole Polytechnique Fédérale de Lausanne (EPFL), Lausanne, Switzerland; Institut de Pharmacologie et de Biologie Structurale, France

## Abstract

We describe the benefit of using reconstructed ancestral sequences (RAS) on resequencing microarrays for rapid pathogen identification, with *Enterobacteriaceae rpoB* sequences as a model. Our results demonstrate a sharp improvement of call rate and accuracy when using RASs as compared to extant sequences. This improvement was attributed to the lower sequence divergence of RASs, which also expanded the sequence space covered by the microarray. Extension of this novel microarray design strategy to viruses, antimicrobial resistance elements or toxins is straightforward.

## Introduction

The emergence of novel pathogens that threaten public health is unpredictable. The 2003 SARS epidemic, with a novel coronavirus variant diffusing widely while its biological identity was still unknown, is a paradigm that illustrates two essential requirements of biothreat preparedness: the ability to identify yet unknown agents, and to do it rapidly. The threat of deliberate release of infectious agents in areas where they are not generally encountered, or the natural evolution of novel combinations of genetic material, exemplified by the H1N1 2009 pandemic variant [1], further stress the need for rapid identification of unexpected agents. Efficient identification platforms must also cope with the large diversity of pathogens and the need to differentiate them from closely related non-pathogenic species [2].

Nucleic acid sequences allow pathogen identification by homology search and phylogenetic positioning, and can achieve species- or strain-level precision. One strategy relies on the amplification and sequencing of conserved target genes, such as bacterial 16S rRNA genes or viral RNA-dependent RNA polymerase genes. Even though broad range primers are used with success in many diagnostic and discovery applications, these approaches are limited in their phylogenetic span, fail to identify species with incompatible sequence variation in priming sites, and do not detect genetic reassortants. High throughput sequencing platforms offer a novel and powerful approach for identifying known or yet unknown pathogenic organisms [3], but the current time to results may still represent a limitation in the event of a public health emergency.

High-density resequencing DNA arrays allow rapid detection of a broad spectrum of infectious agents [2], [4], [5], [6], [7], [8], [9], [10], [11], [12], [13]. One interesting feature of resequencing microarrays is the possibility to detect nucleic acids in a sample, even if their sequence diverges by up to 10–15% from those that are represented on the array [10], [11]. Therefore, even if a novel emerging agent would differ markedly from all known sequences, as was the case for the novel 2003 coronavirus [14], [15], it could be possible to detect it with precision. However, in face of the huge diversity of the microbial world [16], [17], there is a clear need to improve phylogenetic coverage by microarrays. In addition, both accuracy and sensitivity are expected to decrease with increasing levels of sequence mismatch between the microbial agent present in the sample and those represented on the microarray. Given that chip size and density are finite, the number of sequences that one array is able to detect must be increased by optimization of the covered sequence space. One solution is to tile sequences separated by an optimal phylogenetic distance (e.g. 5%). Yet another improvement could consist of reducing the expected divergence between tiled sequences and the sequence of pathogens to be detected.

**Figure 1:**
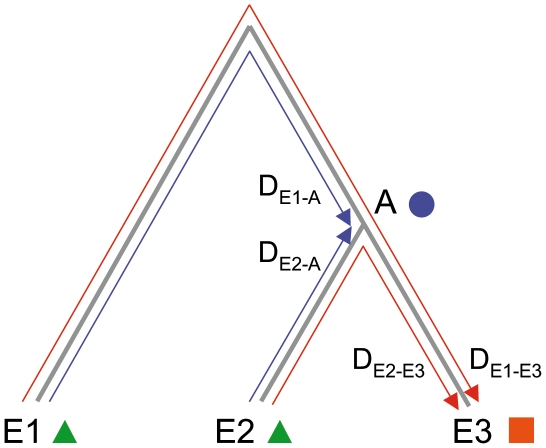
Schematic representation of sequence evolution. The phylogenetic distance of extant sequences to ancestral sequences is reduced, as compared to the distance among extant sequences.

Reconstructed ancestral sequences (RAS) have the desirable property of being more closely related to derived sequences than the latter are among themselves. Clearly, the distance between sequences evolving by a mutational process will, on average, diverge twice as fast between them, relative to their common ancestor (**Fig. 1**). This property was recognized long ago, e.g., [18], and can be exploited in several applications including as seeds in Blast homology searches [19], protein family discovery and functional predictions [20], [21], [22]. Simulation studies have shown that given a phylogenetic tree and an unbiased phylogenetically representative set of extant homologous sequences, ancestral sequences can be inferred with high accuracy provided that evolutionary rate heterogeneity among sites and lineages is incorporated in the evolutionary models [18], [19], [22], [23], [24].

The aim of this study was to demonstrate and assess quantitatively, the advantage provided by using RASs on resequencing DNA microarrays. Although available 16S rRNA gene sequences provide the best current coverage of bacterial phylogenetic diversity, protein-coding genes provide improved resolution (for example, 16S RNA sequences of *Yersinia pestis*, the agent of plague, and *Y. pseudotuberculosis* are identical) and some genes, e.g. *rpoB* coding for the beta subunit of RNA polymerase, can be found in nearly all lineages [25]. In addition, the codon structure of these genes makes their evolution easier to model than 16S rRNA sequences. We therefore used *rpoB* sequences of the taxonomic family *Enterobacteriaceae*.

## Results

### 1. Improved call rate and accuracy using tiled ancestral sequences

Ancestral sequences are more closely related to extant sequences, than the latter are among themselves (**Fig. 1**). For instance, for a gene sequence that evolves by mutational divergence (that is, with no homologous recombination), the last common ancestor (A) of two extant species (E2 and E3) that are separated by genetic distance D will diverge, on average (with variance depending on evolutionary rate homogeneity among lineages) by only D/2 from each of its descendants (D_E2-A_≈D_E2-E3_/2, **Fig. 1**). In addition, the ancestral sequence will be more closely related to extant species that do not descend from A (D_E1-A_<D_E1-E3_, **Fig. 1**). Given that call rate and accuracy of resequencing microarrays depend on divergence between tiled and hybridized sequences [11], we sought to demonstrate and quantify the improvement of microarray performance when tiling ancestral sequences.

**Figure 2:**
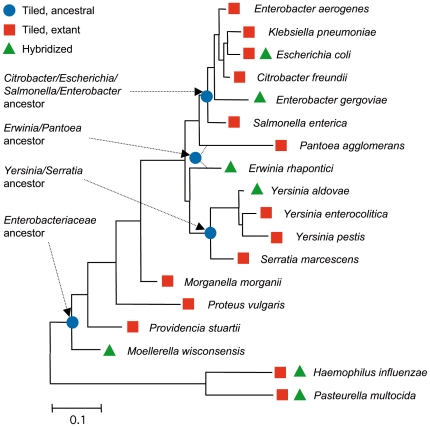
Maximum likelihood phylogeny of *rpoB* sequences used in this study. Reconstructed ancestral sequences and extant strains that were tiled (blue circles) and strains that were hybridized onto the array (red squares) are indicated. Although genera *Pantoea* and *Erwinia* were grouped into a single clade when using the 169 *rpoB* sequences used for inferring ancestral sequences, the *Pantoea*/*Erwinia* clade is not recovered when using this restricted dataset, and the ancestral node was represented with dotted lines connecting the *Pantoea* and *Erwinia* branches.

Gene *rpoB* was sequenced in 169 taxonomic type strains of *Enterobacteriaceae* species and subspecies, representing 43 genera and 169 species [26], [27], [28];(Deletoile, Grimont and Brisse, unpublished). For the purposes of this study, four ancestral nodes were selected at various phylogenetic depths (**Fig. 2**). The most likely *rpoB* sequence of the ancestor of extant lineages that diversified from these four nodes was determined by a maximum likelihood method (**Fig. S1**). As expected, phylogenetic analysis of a combined dataset comprising extant and reconstructed ancestral sequences (RAS) branched the latter at their node with near-zero branch lengths (not shown). The four RASs were tiled on PathogenID v2.0 microarray along with sequences corresponding to 14 extant bacteria (**Fig. 2**).

For hybridization on the array, seven bacterial species were selected: *Enterobacter gergoviae*, *Escherichia coli*, *Yersinia aldovae*, *Erwinia rhapontici*, *Moellerella wisconsensis*, *Pasteurella multocida* and *Haemophilus influenzae* (**Fig. 2**). For example, *Enterobacter gergoviae* was selected to compare results obtained with the tiled sequence of *E. coli*, its closest relative, with results obtained after hybridization on the RAS of the *Citrobacter/Escherichia/Salmonella/Enterobacter* (CESE) ancestor.

**Figure 3:**
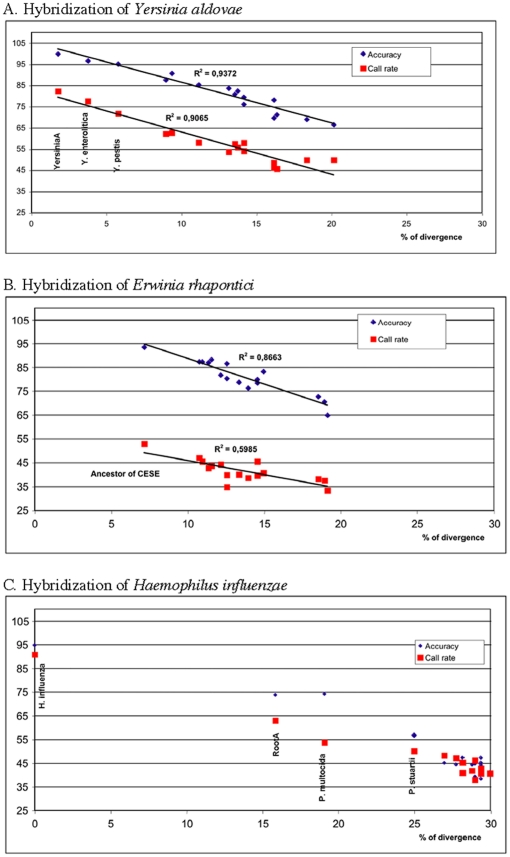
Linear decay of call rate and accuracy with divergence. Plots of call rate (red squares) and accuracy (blue diamonds) values against the percent divergence between tested and tiled *rpoB* sequences. Each panel shows plots obtained for one hybridized strain, as indicated. Tiled sequences with lowest divergence to the hybridized strains are indicated. YersiniaA, ancestral sequence of the Yersinia clade; PErwiniaA, ancestral sequence of the Pantoea/Erwinia clade; CESE, *Citrobacter/Escherichia/Salmonella/Enterobacter* clade; RootA, ancestral sequence of all *Enterobacteriaceae*.

For each of the seven hybridized strains, sequences were obtained from the 18 tiled *rpoB* sequences (**Figure S2**). The call rate and accuracy values for each of the 126 obtained sequences were recorded (**Table S1**) and are plotted against genetic divergence, for three test species, on **Figure 3**. There was a clear linear decay of call rate and accuracy values with divergence. Accordingly, we noted a very sharp increase of call rate and accuracy provided by tiled RASs, relative to extant sequences descending from these ancestors. For instance, when hybridizing *Y. aldovae* total DNA on the array (**Fig. 3A**), the best values for call rate (82.2%) and accuracy (99.75%) were obtained with the tiled RAS corresponding to the ancestor of the *Yersinia/Serratia* clade. The values obtained with close relatives of *Y. aldovae* (*Y. enterolitica* and *Y. pestis*) were slightly lower (77.6/96.5 and 71.7/95.03, respectively). This result is in agreement with the fact that the *rpoB* sequence of *Y. aldovae* diverges from the RAS of the *Yersinia/Serratia* clade by only 1.79%, but by 3.79% and 5.79% from *Y. enterolitica* and *Y. pestis*, respectively.

Likewise, when *Erwinia rhapontici* was hybridized on the array, the best call rate (52.8%) and accuracy (93.25%) values were obtained with the RAS of the *Pantoea/Erwinia* clade, which diverged from *E. rhapontici* sequence by 7.18% (**Fig. 3B**). The next best values were obtained for the RAS of the CESE clade, which was also the next most-closely related sequence. Further, the accuracy value (81.52%) obtained with the RAS of the *Serratia/Yersinia* clade was slightly higher than that obtained (78.31–80.13%) with extant members of this clade (**Table S1**).

Similar results were obtained when hybridizing *M. wisconsensis* and *E. gergoviae*: the values obtained from tiled RAS were higher than those obtained with the tiled sequence of extant members of the clades derived from the ancestor considered (*Enterobacteriaceae* and CESE, respectively; **Table S1**).

These above results show that ancestral sequences improve resequencing results not only for descendants of the tiled ancestors, but also for species that do not descend from these ancestors, when they are members of sister groups that have no representative tiled on the array. To further illustrate this point, we tested hybridization with DNA from *Haemophilus influenzae*, which is not a member of *Enterobacteriaceae*. The RAS of all *Enterobacteriaceae* gave, by far, the best accuracy and call rate values, when compared to tiled sequences of extant *Enterobacteriaceae* members (**Fig. 3C**, **Table S1**). It is remarkable that by using the *Enterobacteriaceae* RAS as tiled sequence, the accuracy jumped from 45% (maximal value obtained with extant *Enterobacteriaceae* members) or 56.8% (value obtained with *P. stuartii*) to 73.7%. The low values obtained with extant *Enterobacteriaceae* members are consistent with their high sequence divergence from *H. influenzae* (25–32%), while the *Enterobacteriaceae* RAS diverged only by 15.9%. The *Enterobacteriaceae* RAS provided similar improvement when hybridizing DNA from *Pasteurella multocida* (**Table S1**).

Clearly, when tiled sequences of some extant members of a clade are less divergent than the RAS of the clade, the RAS is not expected to provide an advantage. This is illustrated for *E. coli*: the tiled sequences of *C. freundii*, *E. aerogenes and K. pneumoniae* are closer from *E. coli* - and accordingly, provided better results (**Table S1**), than the sequence of the ancestor of clade CESE, which also comprises *S. enterica* and *E. gergoviae* (**Fig. 2**).

### 2. Reconstructed ancestral sequences provide increased phylogenetic coverage

**Figure 4:**
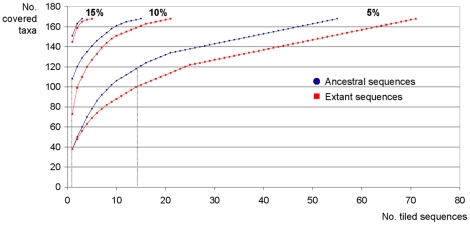
Phylogenetic coverage achieved when using reconstructed ancestral sequences or extant sequences. The graph is based on *rpoB* sequences of 169 *Enterobacteriaceae* taxa. The X-axis gives the number of sequences (blue circles, ancestral sequences; red squares, extant sequences) needed to achieve coverage of the number of taxa on the Y-axis. Curves are given for three levels of maximal divergence tolerated between tiled and hybridized sequences. Vertical dotted lines highlight two comparisons discussed in the text.

As the use of RASs reduces the distance between tiled sequences and those of extant organisms, incorporation of RASs in microarray design should allow reducing the number of sequences that need to be tiled, for a desired coverage of phylogenetic diversity. To quantify this beneficial effect of RASs, we reconstructed ancestral sequences at all nodes of the phylogeny obtained for 169 *rpoB* sequences. We then calculated the number of required RASs to achieve full coverage of extant sequences at divergence levels of 5, 10 and 15%; that is, when each extant sequence diverges by less than the chosen threshold from at least one ancestral sequence (**Fig. 4**). To cover all *Enterobacteriaceae* species with a maximum of 5% divergence, only 53 ancestral sequences are required, while it would be necessary to tile 69 extant sequences. Thus, an economy of 23% oligonucleotide probes required on the array would be achieved. At this divergence level, while 14 sequences of extant species would cover 100 *Enterobacteriaceae* species, 14 RASs would cover 120 *Enterobacteriaceae* species. Likewise, at the 10% divergence threshold, the single extant sequence with the highest coverage would cover 73 species, whereas the single RAS with the best coverage would cover 108 species (a 48% increase). At a 15% threshold, the improvement provided by the use of RASs was more modest (**Fig. 4**).

## Discussion

An important advantage of resequencing microarrays is the possibility of discovering unknown pathogens that harbor sequences that are similar, but distinct, to those of organisms that have been sequenced. This property has allowed detection of emerging strains of pathogens that were not tiled on the array [2], [29], [30]. In addition, as a unique tiled sequence can serve for resequencing several closely related pathogens, this property mitigates an important limitation of microarrays, namely the finite number of probes it can contain. However, we observed a linear decay of call rate and accuracy as a function of sequence divergence, as recently reported for Rhabdoviruses [11], showing that the quality of the signal is dependent on the nature of the sequences incorporated in the microarray design. Even though sequences obtained with tiled sequences that diverge by up to 10–15% still contain useful nucleotide information, it is important for the accuracy of the results that the tiled sequences diverge minimally from the sequences potentially present in the sample. Our results provide a clear demonstration that the use of RASs improves very significantly both the call rate and the accuracy, as expected given the dependency of these parameters on sequence divergence. Further, pathogens that are too divergent (beyond 15–25%), including potential novel emerging pathogens, might be missed by tiled extant sequences but detected when using ancestral sequences. We also reasoned that it should be possible to increase the coverage of extant pathogens by using RASs, while reducing the number of required probes for a desired coverage level. Our results show that the use of RASs would allow minimizing by approximately 25% the number of sequences that need to be tiled on the microarray to cover at 5% divergence, the entire diversity of the taxonomic family *Enterobacteriaceae*. Although the gain provided by ancestral sequences will vary depending on the phylogenetic structure of the group considered, this value show that the use of RASs can broaden significantly the sequence space around the sequences tiled on a microarray.

The *rpoB* sequence dataset used here as a proof of principle had the appropriate properties of good phylogenetic coverage, relatively low maximal sequence divergence and lack of detected horizontal gene transfer or gene mosaicism. Reconstruction of ancestral sequences can in principle be achieved for any set of homologous sequences, including for example viral polymerase sequences. In addition, insertion/deletions among extant sequences can also be incorporated in ancestral sequence reconstructions [24]. However, the accuracy of ancestral sequences is strongly affected by evolutionary phenomena such as homologous recombination and highly heterogeneous evolutionary rate among sites or lineages [19]. This may in particular restrict applicability to 16S rRNA, given the extreme among-sites rate heterogeneity of this gene, as shown in *Enterobacteriaceae* [31]. Accuracy of ancestral sequence reconstruction is also dependent on a good estimation of the tree topology and branch lengths [18]. In the case of our *rpoB* dataset, these parameters were not strongly affected by the tree inference method (not shown). Besides maximum likelihood, other methods, including maximum parsimony, distance and Bayesian methods, are available for ancestral sequence reconstruction [18], [19], [22], [23], [32]. Although it was not the purpose of this work, it could be important to evaluate their relative accuracies, which may vary depending on the specific properties of the set of extant sequences considered [19], [22].

### Conclusions

In this work, we demonstrated that using reconstructed ancestral sequences on microarrays broadens the sequence space targeted by these tools and can therefore facilitate pathogen detection. The use of RAS improved the two major parameters of microarrray resequencing, call rate and sequence accuracy, which have a major influence on the subsequent processes of identification of novel sequences, such as Blast searches in sequence databases or confirmatory experiments based on targeted nucleic acid amplification. Therefore, the use of ancestral sequences should be regarded as an important strategy to improve the design of microarrays aimed at identification of pathogens of public health importance.

## Materials and Methods

### Ancestral *rpoB* sequences reconstruction

The phylogeny used for ancestral sequences reconstruction was based on 169 *rpoB* sequences of *Enterobacteriaceae* type strains [26], [27], [28] (Deletoile, Grimont and Brisse, unpublished), representing 169 distinct species and subspecies belonging to 43 genera. Endosymbiont sequences were excluded given their convergent evolution towards increased A+T content. There was no insertion or deletion in the 501 nucleotides portion considered. A neighbor-joining tree was obtained using software BioNumerics v5.10 (Applied-Maths, Belgium). Ancestral sequences were reconstructed by maximum likelihood using the software PAML v4 [23]. The nucleotide substitution model used was K80 with parameters gamma (number of categories of distinct substitution rates) and kappa (transition/transversion ratio) estimated.

### Content of the “PathogenID v2.0” resequencing microarray

The “PathogenID v2.0” microarray (Berthet et al) was designed to detect a large panel of pathogens by resequencing-based DNA hybridization. 949 sequences, amounting to 300,000 total bp, were tiled on the microarray. The microarray contains a minimum of 2.5 million of 25-mers probes, which were synthesized *in situ* by photolithography [33]. This technology allows re-sequencing of samples on both strands.

The principle of the resequencing array is designed to interrogate each single base with a set of eight 25-mer probes for a specific sequence tiled [34]. Two probes among the eight designed (4 for each sense of the region of the sequence selected, i.e. forward and reverse) correspond to perfect matches at the central (13^th^) position of the probe, while all other probes represent all other possible mismatches at the same position.

The selected sequences cover a large number of genes for viral and bacterial identification as well as genetic elements such as antibiotic resistance genes and major genes involved in toxin production and pathogenicity. For the purposes of this study, a set of 14 sequences corresponding to an internal sequence of gene *rpoB* were tiled on the array. These include the *rpoB* sequence of 12 members of family *Enterobatecriaceae*: *Escherichia coli*, *Citrobacter freundii*, *Enterobacter aerogenes*, *Klebsiella pneumoniae*, *Morganella morganii*, *Pantoea agglomerans*, *Providencia stuartii*, *Proteus vulgaris*, *Salmonella enterica*, *Serratia marcescens*, *Yersinia enterocolytica* and *Yersinia pestis*. The *rpoB* sequences of *Pasteurella multocida* and *Haemophilus influenzae* were tiled as well; these species were selected as members of the gamma-Proteobacteria groups that are most closely related to the family *Enterobacteriaceae* based on 16S rRNA gene sequences [35]. In addition, the four reconstructed ancestral sequences were tiled on the chip. These ancestral sequences corresponded to (i) the common ancestor of all *Enterobacteriaceae*, (ii) the common ancestor of *Yersinia* genus, (iii) the common ancestor of the clade comprising genera *Pantoea* and *Erwinia*, and (iv) the common ancestor of the clade comprising genera *Escherichia*, *Salmonella*, *Citrobacter* and *Enterobacter* (sequences are given as **supplementary material S1**).

### Hybridization to microarrays

DNA of each bacterial strain tested was extracted using the Wizard kit (Promega, France) according to the manufacturer's instructions. Nucleic acid amplification was performed by Repli-g Mini Kit according to Qiagen's instructions. Five micrograms of DNA, quantified by the Quantit kit provided by Invitrogen, were fragmented and labeled using the GeneChip Resequencing Assay Kit (Affymetrix Inc.), hybridized overnight at 45°C and washed, stained and scanned according to manufacturer's instructions (Affymetrix, Inc. Santa Clara, CA).

### Microarray data analysis

After the scan of the microarray, the raw image file (.DAT) is transformed using GeneChip® Operating Software (GCOS) (Affymetrix Inc.) to a fluorescence intensity file (.CEL). Bases are called by the GeneChip® Sequence Analysis Software (GSEQ) which uses a derivative of the ABACUS base-calling algorithm [36]. Sequences are outputted in FASTA format.

### Sequence analysis

We used BioNumerics v5.10 (Applied-Maths, Belgium) software to calculate the percentage of divergence between *rpoB* sequences. The call rate value was defined as the ratio of the number of determined bases to the sequence length. The accuracy of the microarray resequencing process was defined as the ratio of the number of correctly determined bases to the total number of determined bases, by comparison with the known *rpoB* sequence of the tested strains.

### Coverage of diversity by extant and ancestral *rpoB* sequences

To predict the phylogenetic coverage of ancestral sequences, we computed for each ancestral sequence, the number of extant sequences that diverged from it by <5%. Once this was calculated for each of the 169 ancestral sequences, ancestral sequences were ordered by the number of covered (<5% divergence) extant sequences. The ancestral sequence with the highest number was selected and the corresponding number of extant sequences was recorded (first value on the Y-axis, **Figure 4**) and the covered extant sequences were removed. This process was reiterated for all ancestral sequences by decreasing order of covered extant sequences. The same process was performed using 10% and 15% thresholds. To compare the above results with the coverage obtained with extant *Enterobacteriaceae* sequences, we collected the same data by comparing the extant sequences among themselves.

## Supporting Information

Figure S1 The four reconstructed ancestral *rpoB* sequences that were tiled on the PathogenID resequencing microarray.
(DOC)

Figure S2 The 126 raw sequences obtained after hybridization of seven tested strains on the PathogenID resequencing microarray.
(DOC)

Table S1 Call rate and accuracy values obtained after hybridization of seven strains on each of 18 sequences tiled on the PathogenID resequencing microarray. The divergence corresponds to the uncorrected nucleotide sequence divergence between the *rpoB* sequence of tested strains and tiled sequences.
(DOC)
