# Supplementary material for: Reconstructed Ancestral Sequences Improve Pathogen Identification Using Resequencing DNA Microarrays
Source: PLoS One. 2010 Dec 20;5(12):e15243. doi: 10.1371/journal.pone.0015243 (PMC3004854; doi:10.1371/journal.pone.0015243)
Supplement: Figure S1 — The four reconstructed ancestral rpoB sequences that were tiled on the PathogenID resequencing microarray. (DOC) [file pone.0015243.s001.doc]

**Figure S1**. The four reconstructed ancestral *rpoB* sequences that were tiled on the PathogenID resequencing microarray.

>rpoB_root_Ancestral

ACTCACTATGGTCGTGTATGTCCAATCGAAACCCCTGAAGGTCCAAACATCGGTCTGATCAACTCACTTTCTGTTTATGCACAGACTAATGAGTATGGTTTCTTAGAAACACCATATCGTTTAGTGCGTGATGGTCTAGTTACTGATGAAATTCATTACCTGTCTGCAATTGAAGAAGGTAACTTCATCATTGCACAGGCAAACACCGTATTAGATGAAGATGGCCGTTTTATTGATGAACTGGTTACTTGTCGTAACGAAGGTGAATCTAGCTTATTCAGCCGTGAACAGGTTGAATATATGGACGTTTCGACACAACAGGTGGTTTCTGTTGGTGCTTCATTGATCCCATTCCTTGAACACGATGATGCGAACCGTGCATTGATGGGTGCGAACATGCAACGTCAAGCAGTTCCAACATTACGTGCTGATAAACCATTAGTTGGTACAGGTATGGAACGTGCAGTCGCAGTTGACTCCGGTGTTACGGCTGTTGCAAAA

>rpoB_Yersinia_Ancestral

ACTCACTACGGTCGCGTATGTCCAATCGAAACGCCAGAAGGTCCAAACATCGGTCTGATCAACTCCTTGTCTGTGTACGCACAGACCAACGAGTATGGTTTCCTGGAAACCCCTTATCGTCGCGTGCGTGATGGTGTGGTGACCGATGAAATTAACTATCTGTCTGCTATTGAAGAAGGCAACTTCGTTATCGCTCAGGCGAACTCCAACCTGGATGATGAAGGCCGCTTCATGGAAGACCTGGTCACTTGTCGTAGCAAAGGCGAATCAAGCCTGTTCAGCCGCGATCAAGTTGACTACATGGACGTTTCCACTCAACAGGTCGTGTCCGTTGGTGCTTCTCTGATTCCATTCCTGGAACACGATGACGCCAACCGTGCATTGATGGGTGCGAACATGCAACGTCAGGCGGTTCCTACTCTGCGTGCTGATAAGCCGCTGGTGGGTACCGGTATGGAACGTGCGGTAGCGGTTGACTCAGGGGTAACCTCTGTAGCCAAA

>rpoB_Pantoea_Erwinia_Ancestral

ACTCACTACGGTCGCGTATGTCCAATCGAAACGCCGGAAGGTCCGAACATCGGTCTGATCAACTCCCTGTCTGTGTATGCACAGACCAACGAGTATGGTTTCCTGGAAACCCCGTATCGTCGCGTGCGTGACGGTGTGGTGACCGACGAAATTCATTACCTGTCTGCTATTGAAGAAGGTAACTTCGTTATCGCTCAGGCGAACACCAACCTGGACGACGAAGGCCGCTTCGTTGACGATCTGGTTACCTGCCGTAGCAAAGGCGAATCCAGCCTGTTCAGCCGCGATCAGGTTGACTACATGGACGTTTCCACCCAGCAGGTGGTTTCCGTCGGTGCGTCCCTGATCCCGTTCCTGGAACACGATGACGCCAACCGTGCATTGATGGGTGCGAACATGCAACGTCAGGCGGTTCCTACTCTGCGTGCTGATAAGCCGCTGGTTGGTACCGGTATGGAACGTGCTGTTGCGGTTGACTCCGGTGTAACCGCCGTAGCCAAA

>rpoB_Citro_Salmo_Escherichia_Enterobacter_Ancestral

ACTCACTACGGTCGCGTATGTCCAATCGAAACGCCTGAAGGTCCGAACATCGGTCTGATCAACTCCCTGTCCGTGTACGCACAGACTAACGAATACGGCTTCCTTGAAACCCCGTATCGTAAAGTGACTGACGGTGTGGTGACCGACGAAATTCATTACCTGTCTGCTATTGAAGAAGGCAACTTCGTTATCGCTCAGGCGAACTCCAACCTGGATGACGAAGGCCGCTTCGTAGAAGATCTGGTTACCTGCCGTAGCAAAGGCGAATCCAGCCTGTTCAGCCGCGACCAGGTTGACTACATGGACGTTTCCACCCAGCAGGTGGTTTCCGTCGGTGCGTCCCTGATCCCGTTCCTGGAACACGATGACGCCAACCGTGCATTGATGGGTGCGAACATGCAACGTCAGGCGGTTCCGACTCTGCGCGCTGATAAGCCGCTGGTTGGTACCGGTATGGAACGTGCTGTTGCCGTTGACTCCGGTGTAACCGCCGTTGCCAAA
