# Supplementary material for: Reconstructed Ancestral Sequences Improve Pathogen Identification Using Resequencing DNA Microarrays
Source: PLoS One. 2010 Dec 20;5(12):e15243. doi: 10.1371/journal.pone.0015243 (PMC3004854; doi:10.1371/journal.pone.0015243)
Supplement: Figure S2 — The 126 raw sequences obtained after hybridization of seven tested strains on the PathogenID resequencing microarray. (DOC) [file pone.0015243.s002.doc]

**Figure S2**. The 126 raw sequences obtained after hybridization of seven tested strains on the PathogenID resequencing microarray.

>seq_tiled_Cfreundii_seq_tested_Erhapontici

CGCGNATGTCCAATCGAAACGCCGGNNNNNNNANACATCGGTCNGATCAACNGGTNNNNNNNTNNNNNNNCNNNNNNNNNNNNCNNCNNNANNNCCNNNNNNCGNNGNGNNNCNGNNGNNNNCNNNGNNNNNNNCGAAATTCATTNCNNCNNNGNNNNNNNNNNCNNNGGNNNCGNNATCGNTCNGGCGNANANNNNNNNCNNNNNNGNNNNCNNNNNNGNNGNNNNNNNNCNGNCCTGCCGTAGCAAAGGCNNNNCNNNNNCTNNNCGNNGNNNNNNGNTTGACTACATGGACGNTNNNNGNNNGNNNNNNNNTNNNGNCGNTNCGNNNNTGATNNCGNNNCTGNNGNNCNNNGNCGNNNNCCNCGCATTGNNNNNNGCANNNNNGCANCGNCNGGCGGNTNNANNNNNNGNNNNNGNNNNGCCGCTGGTTGGNGNTGNNNNNNNNNNNGNNNNNNNNGNNNNNNNCGNNGNNNNN

>seq_tiled_Cfreundii_seq_tested_Ecoli

CGCNNNNGTCCAATCGNANCNCCTGAAGNTCCGAACATCGGTCNGNNCAACTCCCTGTCCGTGNACGCACAGACTAACGAATACGGCTTCNNTGAGACNCCGTATCGTAAAGTGACCGACGNTGTTGTAACTGACGAAATNCNCNGTNGGNCTGCTATCGAAGAAGGCAACTACNNTATCGNNCNGGCGAACTCCAACCTGGATGAAGNCCNNGGNNNCGNNNNAGATCTGGTNNTCTGNCGTAGCAAAGNCGAATCCAGNNTGTTCNGCNGCTACCAGGTTGACTACATGGACGTATCCANCCAGCAGGNGGTNTCCGTCGGTGCGTNNNTGATNCCGTTNCTGNNNNACGNNGNCGNNNACCGTGCATTGATNGGTGCGAACANGNANCNNCNGNCCNTTNNGNNNCTGNNTCNCGNNNNGCCGCTGGTTGGTNNGGGNNTGGAACGTGNNGNTGNCGNTGANTCCGGTGTANNN

>seq_tiled_Cfreundii_seq_tested_Egergoviae

CGNGNCNNCNNNNNCNNNNCNNCGNNNNGTCCGAACATCGGNCTGATCAACTCCCTGTCCGNGNNCGCNCAGACTAACGAATACGGCTTCNNNCCNNNGNNNCGTCGNAAAGTGNCCGNNGNCNNGNNNGNNNGCGAAATTCATTACCTGNCNGCNGNTGNGNNCNNNNNNNNNNGCNNNNNNNCNGCGAACTCCAACCNNNCCNNCNNCCNNGGNNNNGGNNNCNNNCTGGTTACCTGCCGNAGCAAAGGCNNNNNGGNNNCNNNNNNNNGCNNCNNGGNTGACTACATGGACGTATCCNNNNNGNNNNCNNNTNNNGNCGGNGCGNNNNTGATNCCGTNCCTGNNNNACGNNGNCGCNNACCGTGCATTGATGGGTGCGAACANGCANCGTCNGNCGNNNNNGNNNNNNNNNNNCGNNGNNNNGCNGNNNNGNGCCGGTATGGAACGTGCNNNNGNNGNNGNNGNNNNCNNNGNN

>seq_tiled_Cfreundii_seq_tested_Hinfluenzae

GGNNANNCNNAANNNACNNCCNNTNAAGGTCCANACANCGNTTAGATCNNNNNNGNTNNNNCNNNNNNNGCANNNNNNCCNNNTNNNNNNANNNNNNNGGNNCGNCGNNANNNNNNNNNNCNNNNNCNGGNNGCNCNNNNNNNACGGGNNNGGNGAAANCANCNNNGNNNNNNNNNNNNNNNNNNNNCNNTNATNNANNNNNNNNNNNNCCNNNGNGNNNNNNNTANNNNTNNNGNNNGNNGCNNNCNNANNNAANNNNNGNNNTGTNGNNNCNTGGNNNNGNNNNNNNCNNNNNNTNNAGNNNNNNNNNCNNNTNTNATNNNCNNNGGGNNNNNNNNNNNGNNNCNGNANAANNCAGNNNNANGTGCGTTGATGGGTGCGAACANGCAACGNGAANNAANNTNANNNNNNNNNNNCNGNNNNNNGTCNNNNNNNNNAGNNNTTNNTNNTNNGNANGGGNNTNNNGGANCCNNGNTN

>seq_tiled_Cfreundii_seq_tested_Mwisconsensis

NGNNNGNGTCCAATCGAAACGCCAGAANGTCCANACATCGGNCNGATTANNNNGNNNNNNNNNNNNNNNCAAACNNNNNNNNNNNNCNNNANNNNNNNNGNNCGGNNCNANNNNGNNNNTNNCNNNNNAACTGACNNNTCNCATTACCTGTCNGNGNNTNNNNCNNANNNNNNNANNNNGNNNNNNNNNNNNNTNNNNNTGGATGACGANCNNNGNNNNNNNNCNNNANNNNNNGGNCNNNNCNCNNNNANCNNNNNNNNNNNNNNTNGCNGCNNANNNNNNNNNNNNNNNNNNNNTNNNNNNNNNNNNNNNNNTNTGNNNNNNNNNGTGNNGNTNNNGNNNCNTCCNANNNNNNCTGNTNNCCGTGCATTGATNGGTGCGAACANGCAACNTCAGNCTNCNGNNNTNNNNNNNNNCNNNNNNNNGNNANANNNNGTAGGTATGGANNGNGNGNNNNNNNNTNNNNGNNNNNNNGTN

>seq_tiled_Cfreundii_seq_tested_Pmulticoda

NNTGNCNGTCCAATCGAAACGCCTGAAGGTCCANACANTNCGTNANNNCNNNNGGTGTCCGNNNCNGNNNCANNNTNCNNGNNCNCCNNGANNANNNNGNNNCGTNNNNANNNGNNNNGNNTNNNNNANGAGGGNNNNCNNGNANGNGNNCNNNNNGNTNANNNNCNCGTNNTTNTNNTCGCTCAGGCGAANTGGNANGNNNNNNNNNNNCCNNNNNNNNCCNCNNNAGCCNNNNAGNGNNGCGNNNNNACCANNNNNCNCNTGTTTNGCCGNTNTNNTCTTGNNNCNGNNNNNNNTNNNNGANNNNGNCNNNNTNNNGNCGNTGCGNGGGNNNNGGNNNNTTNTCNGTNCGNCCTNGNGNNANNNGNGATANTCNNNGCANACATGCANCGTCAAGCGNTNNNNGTNTNNNNCNNCGANNNGNNANNNANNNNAGTCGGNNNNNNTANTGCTGTTGNCGNNGCNGGTNNCNANGNN

>seq_tiled_Cfreundii_seq_tested_Yersinia_aldovae

CGCGNNNGTCCAATCGAAACGCCAGAAGGTCCANACATCGGNCNGATTAACNNNCTGNNNNNNNNNNNNNCACNCNACNNTNNCNCNNNGNNNNNNNTGNNNNGNNACNNNNNGGNNGNCNCCANNGNNCNNGGCCNNNANNNNNGTNGNNNNGCTATTGAAGANNGCANNNTCGTTATCGNTCAGNCGAACNCCAACCTGGATGATGCCCNNNGNNNGNCNGAAGANCNGNNNGTNNGTCGNNGCAAAGGCGANNCNNNNNNNGNGNGNNGCGNTNAGGTTGACTATATGGNCNNNNNNGGGNNNNNGNCNTNNNNGNNNGNNNNNGNNNNNTNGGNNCGNCTGGAACACGANGNCGCNNACCGNGCATTGATNGGTGCGAACANGCAACGTCAGGCGGTTNNTNNTNNNNNTNNCNNNGNNNNGTCNNNNNNNNNCGGTATGGAACNNGNGNGNNGNNNNNNTGGNNNCNNNGTN

>seq_tiled_CitSalEscEntA__seq_tested_Erhapontici

CGCGNATGTCCAATCGAAACGCCGGNNNNNNNANACATCGGTCNGATCAACNGGTNNNNNNNTNNNNNNCNNNNCNNNNNNNNCNNCANNNNNNNNNNGGGNCNNNNCGNNNNNGNNGGNNNCNNGNNGANCGNCGAAATTCATTNCCNCNCNGCGNNNGNNNCNNNNNNNNNNNGNNNCGGNCNGGCGNANANNNNNNNCNNNANTNNTNNNGNNNGNNNNNNCNTNNNNCNGNCCTGCCGTAGCAAAGNCNAANNNNNCNNNNNCNNCNNNNNNCNNNTTGACTACATGGACGTTTNCNNNCAGCNGNNGGTTNNNGNCNGTGCGNNNNTGATNNCGNNNCTGNNGNNCNNNGNCGNNNNCCNCGCATTGNNNNNNGCANNNNNGCANCGNCNGGCGGNTNNANNNNNNGNNNNNGNNNNGCCGCTGGTTGGNGNTGNNNNNNNNNNNGNNNNNNNNGNNNNNGNNNNNNNGGNN

>seq_tiled_CitSalEscEntA_seq_tested_Ecoli

CGCNNNNGTCCAATCGNANCNCCTGAAGNTCCGAACATCGGTCNGNNCAACTCCCTGTCCGTGTACGCACAGNCNAACGAANACGGCTTCCTNNNGANTGNGTATCGTAAAGTGNCCGNCNCCNNNNNNNNNGNNNNNCNCNNCNGTNNNNTNGCNNTCGAAGNNGGCAACNACGNNNNNNNCCNGGCGAACTCCAACCTGGANGNANCNNNNNANGGNNNNNNNNANCTGGTNNTCTGNCGTAGCAAANNCGAATCCNGNNNNTTCNNCCNCGANCNGNTTGACTACATGGACGNANNNNGNCNNCCNNCGNNNNNNGNCGGTGCGTNNNTGATNCCGTTNCTGNNNNACGNNGNCGNNNACCGTGCATTGATNNGTGCGAACANGNANCNNCNGNCCNTTNNGNNNCTGNNTCNCGNNNNGCCGCTGGTTGGTNNGGGNNTGGAACGTGNNGNTGNCGNTGNNNCCGGTGNNNNN

>seq_tiled_CitSalEscEntA_seq_tested_Egergoviae

CGNGNCNNCNNNNNCNNNNCNNCGNNNNGTCCGAACATCGGNCTGATCAACTCCCTGTCCGTGTNCGCACAGACTAACGAATACGGCTNNNNCGAAANCNNGTATCGTAAAGNGNNCGNCGNCGNGNNGNCCGNCGAAATTCATTACCTGTCTGCTATTGANNNNNNNNNNNNNNGCTNTNNNNNNGCGAACTCCAACCNNNCCNNNNNNNNNNGNNGNGNNNNCNATCTGGTTACCTGCCGNAGCAAAGNCNNNNCGNNCNNGNTCAGCNGCGNCCNGNTTGACTACATGGNCGNANNNGGNNNGCNGGNGGTTNNNGNCGNNGCGNNNNTGATNCCGTNCCTGNNNNACGNNGNCGCNNACCGTGCATTGATGGGTGCGAACANGCANCGNCNGNCGNNNNNGNNNNNNNNNNNCGNNGNNNNGCNGNNNNGNGCCGGTATGGAACGTGCNNNNGNNGNNGNNGNNNNCNNGGNC

>seq_tiled_CitSalEscEntA_seq_tested_Hinfluenzae

GGNNANNCNNAANNNACNNCCNNTNAAGGTCCANACANCGNTTAGATCNNNNNGGNTNTNNNTANNNNNNCACANNNNCCNNNTNNCNNGNNNCNNNNGGNANNNCNCNNANTNNGNGGNNTNNNNNCGNTGGNCCCANNTNNACGGGNNCGGNNGGNNGANNANGGCAANNNGNNNNNNTNNNNNNCNNTNATNNANNNNNNNNNNNNTNNNNNNGNNNNNNNTANNNNTNNNGNNNGNNGCNNNCNNANNNNANNANANNNTTTTNGCNGCNCNNNNNNNNNANGNGNTCNCNNTNNNGNGNNNCCNNNNNTTTNNNNTTTCNNNGGGNNNNNNNNNNNGNNNCNGNANAANNCAGNNNNANGTGCGTTGATGGGTGCGAACANGCAACGNGAANNAANNTNANNNNNNNNNNNCNGNNNNNNGTCNNNNNNNNNAGNNNTTNNTNNTNNGNANGGGNNTGCNGNNNNNNNNNNN

>seq_tiled_CitSalEscEntA_seq_tested_Mwisconsensis

NGNNNGNGTCCAATCGAAACGCCAGAANGTCCANACATCGGNCNGATTANNNNNNNGTCTGNNANCGCACAAACNNNNNNNNNNNNCNNNNNNNNNNNNNGNNNNNNNGNNNNNNNNGNNNACNNNNNNNGNNNNNNNCCNCATTACCTGTCTGCGNTTGAANNNNCNNNNNNGNNCNNGNNNNNNNNNNNNNTNNNNNTGGATGANNNNNNNNGNNNNNNNNNTNNNNNNNNNGGNCNNNNCNCNNNNANCNNNNNNNNNNNNNTNNNCNGCNCNNNNNNNNNNNATNTGGNCNNTNNNNGNNNNNNNNNNNTTNNNGNNNNCNNTGTGNNGNTNNNGNNNCNTCCNANNNNNNCTGNTNNCCGTGCATTGATNGNTGCGAACANGCAACNTCAGNCTNCNGNNNTNNNNNNNNNCNNNNNNNNGNNANANNNNGTAGGTATGGANNGNGNGNNNNNNNNTGACTCCGGTGTNNTN

>seq_tiled_CitSalEscEntA_seq_tested_Pmulticoda

NNTGNCNGTCCAATCGAAACGCCTGAAGGTCCANACANTNCGTNANNNCNNNNGGNGTCCGTNTCNGCNNCANTNTNCNNGNNNNNCNNNNGNCNCNNNGNANATCNTNNNNNGNTNAGCGNNNNNNNGNNGNNNNCCNNNGNANGNGNTCTNGCGATTNAANANGNCAANNTNGNCANCGCTCAGGCGAANTGGNANGNNNNNNNNNNNCNNNNNNGNNCTNCCANAGCCNNNNAGNGNNGCGNNNNNACCACNNCNANCTNNTTTNNCCACCNNGNCNNTCGTNNTGNCNGCNTTNANNGNANNCNNNNNGTTNNNNNNNNTGCGNGGGNNNNGGNNNNTTNTCNGTNCGNCCTNGNGNNANNNGNGATANTCNNNGCAAACATGCANCGTCAAGCGNTNNNNGTNTNNNNCNNCGANNNGNNANNNANNNNAGTCGGNNNNNNTANTGCTGTTGNCGNNGNNNNTNNTNNNGTC

>seq_tiled_CitSalEscEntA_seq_tested_Yersinia_aldovae

CGCGNNNGTCCAATCGAAACGCCAGAAGGTCCANACATCGGNCNGATTAACNNNNNGNNNNNNNNNNNNNCNNNCNACNNTNNNNCCNNGNNNNNNNAGGNNNNNNNCGNNNNGNCNGNNNNNGTGGTGACNGNTNCNNANNNNNGNNNNNCTGCTATTGAAGAAGGCANCNTNGTNATCNNTCAGNCGAACNCCAACCTGGATGATGATNGNNGNGGNNNNNAAGANCNGNNNGTNNGTCGNNGCAAAGGCGAANCANNCNANNNNNNCNGCGATCAGGTTGNCTNTATGGACGTNGNNGGNNNNCNGNNNNTNGNGNNNNNNNNNGNNNNNTNGGNNCGNCTGGAACACGANGNCGCNNACCGNGCATTGATNGGTGCGAACANGCAACGTCAGGCGGTTNNTNNTNNNNNTNNCNNNGNNNNGTCNNNNNNNNNCGGTATGGAACNNGNGNGNNGNNNNNNTGNNNNTNNGGNC

>seq_tiled_Eaerogenes__seq_tested_Erhapontici

CGCGNATGTCCAATCGAAACGCCGGNNNNNNNANACATCGGTCNGATCAACNGGTNNNNNNNTNNNNNNCNNNNNNNNGNNNNNNNCNNNGGNNCNNNGNNNCGNNGNGNNNNNNNNGNNCNCNNNNNGGNCGACGAAATTCATTNCNNCNNNGNNNNNGNNNANGNCNNNNNNNGNNNNNNNNNGNCGNANANNNNNNNNNNNNNNGNNNNCNNNNNNNNNNCNNNGGCNNNGGNNNGNCGNNGCAAANNCNCNNNNNNNNCTNNCNNCNGNNNNCNNNTTGACTACATGGACGNTNNNNGNNNGNNNNNNNNTNNNGNCGNTNCGNNNNTGATNNCGNNNCTGNNGNNCNNNGNCGNNNNCCNCGCATTGNNNNNNGCANNNNNGCANCGNCNGGCGGNTNNANNNNNNGNNNNNGNNNNGCCGCTGGTTGGNGNTGNNNNNNNNNNNGNNNNNGNNNCNNNNTCCGGTGTNNNN

>seq_tiled_Eaerogenes_seq_tested_Ecoli

CGCNNNNGTCCAATCGNANCNCCTGAAGNTCCGAACATCGGTCNGNNCAACTCCCTGTCCGTGTACGCACAGNCTAACGAATACGGCTTCNNNNCNNNNGNGNNTCGNAAAGTGACCGACGGNNTTGNANNTGNCNCNNNNNNCNNTNNGNCTGCTATCGAAGAAGGCNNNNGNNNNGNNNGNNNGGCGAACTCCAACCTGGATGAAGNCCNNGGNNNNNNNGAAGATCTGGTNNNCTGCCGNNGCAAAGGCGAATCCAGNNNNTTCNNCNNCGANCNGNTTGACTACATGGACGTATCCANCCAGCAGGNGGTNTCCGTCGGTGCGTNNNTGATNCCGTTNCTGNNNNACGNNGNCGNNNACCGTGCATTGATNNGTGCGAACANGNANCNNCNGNCCNTTNNGNNNCTGNNTCNCGNNNNGCCGCTGGTTGGTNNGGGNNTGGAACGTGNNNNNNCNNNTGNNTCCGGNGNANCT

>seq_tiled_Eaerogenes_seq_tested_Egergoviae

CGNGNCNNCNNNNNCNNNNCNNCGNNNNGTCCGAACATCGGNCTGATCAACTCCCTGTCCGTGTNCGCACAGACTAACGANNNCGCCNGNNNNNNNNNGGGNNGTNGNAAAGTGNCCGNNGNCNNGNNGGNCGNCGAAATTCATTACCTGNCNGNNGNTGNNANNNNNNNNNGNNGNNNCNNCNNGGCGAACTCCANCCNNNNCNNCNNCCNNGGGNNNNNCNNCNNNCTGGTTNCCTGCCGNNGCNANNNCNCNNNGGNNNCNNNCANCNGCGNCCNGNTTGACTACATGGACGTATCCNNNNNGNNNNCNNNTNNNGNCGGNGCGNNNNTGATNCCGTNCCTGNNNNACGNNGNCGCNNACCGTGCATTGATGGGTGCGAACANGCANCGNCNGNCGNNNNNGNNNNNNNNNNNCGNNGNNNNGCNGNNNNGNGCCGGTATGGAACGTGNNGNTGNCGNNGNNNNNNNCTNNGNN

>seq_tiled_Eaerogenes_seq_tested_Hinfluenzaee

GGNNANNCNNAANNNACNNCCNNTNAAGGTCCANACANCGNTTAGATCNNNNNGGNTNTNNNTANNNNNNCACANNNNCCNTNNGNNNNNNNNNCNNNGGNNNGNNNNNANNNNNNNNNTCNNNNTNNNGNNNNNCCNNNNNNACGNGNNCGGNGANNCNAATAAGGNCNAANNAGNNNTGCANGANCANTNATNANNNNNNNCNNNNCCCNNGGNGTTNCCNNNANGNNNNNNGNNNGGNNCNTNNANGNANAANNNNNNNNNNTNNGNNGCNCNNNNNNNNNNNNNNCNNNNNNTNNAGNNNNNNNNNCNNNTNTNATNNNCNNNGGGNNNNNNNNNNNGNNNCNGNANAANNCAGNNNNANGTGCGTTGATGGGTGCGAACANGCAACGNGAANNAANNTNANNNNNNNNNNNCNGNNNNNNGTCNNNNNNNNNAGNNNTTNNTNNTNNGTANNGNACTCCNNNAGGNNTNNNN

>seq_tiled_Eaerogenes_seq_tested_Mwisconsensis

NGNNNGNGTCCAATCGAAACGCCAGAANGTCCANACATCGGNCNGATTANNNNNNNGTCTGNNANCGCACAAACTNACGNANGTGNCNNGNNNNNNNNGNNNNGNNNNNANNNGGNNGNNNNNNNANNNGNNNNNNNNCCNNATTACCTGTNNGCGNNTGAANNNNNNNNANNNANNNNNNNAGCNNCNNANNTNNNNNTGGATGACGANCNNGGNNNTNNNNNCNGANNNNNNGGNNGNNNAGANNNNANCNNNNNNNNNNNTNTNTNCNGNNCNNNNNNNNNANNNNNNNNNNNTNNNNNNNNNNNNNNNNNTNTGNNNNNNNNNGTGNNGNTNNNGNNNCNTCCNANNNNNNCTGNTNNCCGTGCATTGATNGNTGCGAACANGCAACNTCAGNCTNCNGNNNTNNNNNNNNNCNNNNNNNNGNNANANNNNGTAGGTATGGANNGTGNGNNNNNNACTNNTNNNNNNNNNGNN

>seq_tiled_Eaerogenes_seq_tested_Pmulticoda

NNTGNCNGTCCAATCGAAACGCCTGAAGGTCCANACANTNCGTNANNNCNNNNGGNGTCCGTNTCNGCNNCANTNTNCNNCTGCGGNTGGNNNNCCNTNNCANGTCGNNAANNGNGNNNAATNTTTGGNGNNGNNNCCNNGNNANGCGNTCNNGNNGNTNNCNNNCNNGTTNNNNANTNCNCNNAGGCGAANTNGNNNGNNNNNNNNNNNNNNGGNNNGNNCNNNANNGNCNNCNAGNGNNTNCNNNAAGGCNAANANCNNTTGTTNNGCNNNCNNGNCNNTCGTNNAGNNNNNNNTNNNNGANNNNGNCNNNNTNNNGNCGNTGCGNGGGNNNNGGNNNNTTNTCNGTNCGNCCTNGNGNNANNNGNGATANTCNNNGCAAACATGCANCGTCAAGCGNTNNNNGTNTNNNNCNNCGANNNGNNANNNANNNNAGTCGGNNNNNNTANTGTNTNTNNNNCNCNTNNTNNNTNGGNN

>seq_tiled_Eaerogenes_seq_tested_Yersinia_aldovae

CGCGNNNGTCCAATCGAAACGCCAGAAGGTCCANACATCGGNCNGATTAACNNNNNGNNNNNNNNNNNNNCNNNCTNCGATTNCGGNTNNGNNNCNNNGGANNGNNACGNNNNGGNNGNNGNNNNNNNTNNNGNNCCNNANNCNNGTNNNNNNGCNATTGNNCNNNNCTNNNNNACNNANGCTCAGGCGAACNCCAACCTGGATGATGCCCNNNGGGNNNNNNCTNNNTNGNCGGTNNGNNGCNNCNNNNNCACNNNNNNNNNNNNNNANNGCGATCAGGTTGNCTATATGGNCNNNNNNGGGNNNNNGNCNTNNNNGNNNGNNNNNGNNNNNTNGGNNCGNCTGGAACACGANGNCGCNNACCGNGCATTGATNGGTGCGAACANGCAACGTCAGGCGGTTNNTNNTNNNNNTNNCNNNGNNNNGTCNNNNNNNNNCGGTATGGAACNTGNNTGNNGNNCNNNNGNAGGNNNNNTN

>seq_tiled_Ecoli__seq_tested_Erhapontici

CGCGNATGTCCAATCGAAANGGGGGNNNNNNNANACATCGGTCNGATCAACNGGTNNNNNNNTNNNNNNCNNNNCNNNNNNNNCNNCNNNNNNCNNNNGNNNCGNNNNGNNNNNNNNGNTCNCNNNNANNNNNNNNNCNNNCNNNNCNGNNNNGCNGNNNNNNNNNNNGGANNNNNCNNNNGNCNCCNNNNNNNNNNNNNNGCTGANNNCCNNNGGNGNNCNNCCNNGNNNNNGTNNNNNCGNAGCAAAGGCNNNNNNNNNNCTNNCNNCNGNNNNCNNNTTGACTACATGGACGNTNNNNGNNNGNNNNNNNNTNNNGNCGNTNCGNNNNTGATNNCGNNNCTGNNGNNCNNNGNCGNNNNCCNCGCATTGNNNNNNGCANNNNNGCANCGNCNCNNGNNNNNANNNNNGCNNGNNGANNNNCCGCTGGTTGGTNCTGNNNNNNNNNGNGNNNNNNNNGNNNNNGGNNNNNNNNNN

>seq_tiled_Ecoli_seq_tested_Ecoli

CGCNNNNGTCCAATCGAAANNNNTGAAGGTCCGAACATCGGTCNGNNCAACTCCCTGTCCGTGTACGCACAGNCNAACGAANACGGCTTCCTTGAGACTCCGTATCGTAAAGTGACCGACGNTGTTGNAACTGNCGAAATTCACTATCTGTCTGCTATCGAAGAAGGCAACNACGNNATCNNNCAGNCGAACTCCAACCTGGATGAAGAAGGCCACNTCGTAGAAGACCTGGTAACTTGCCGNAGCAAAGNCGAATCCAGNNNNTTCNNCNNCGANCNGNTTGACTACATGGACGTATCCANCCAGCAGGNGGTNTCCGTCGGTGCGTNNNTGATNCCGTTNCTGNNNNACGNNGNCGNNNACCGTGCATTGATNNGTGCGAACANGNANCNTCANNCCGTTCCGACTCTGCNTGNNGANNNGCCGCTGGTTGGTACGGGTATGGAACGTGNNGNTGNCGNTGNNNCNGGTGNNACN

>seq_tiled_Ecoli_seq_tested_Egergoviae

CGNGNCNNCNNNNNCGAAACNNNGGNAGGTCCGAACATCGGNCTGATCAACTCCCTGTCCGTGTNCGCACAGACTAACGAATACGGCNNNGNNCCNNNGNGGNATCGTNAAGTGNCCGNNNNCNNGNNNNNNCNNNNNNNNNNTNNCNGNNNNGNNGNTGNNNNCNNCNNNNNNNNCNNNNNNCNNNCGAACNCCANCCNNNCNNNNACNCNNGGNNNNNNNNNNNNNNNNNNGNNNCNNNGNAGCAAAGGCNNNNNGGNNNCNNNCANCNGCGNCCNGNTTGACTACATGGACGTATCCNNNNNGNNNNCNNNTNNNGNCGGNGCGNNNNTGATNCCGTNCCTGNNNNACGNNGNCGCNNACCGTGCATTGATGGGTGCGAACANGCANCNNCNNNNGNNNNNNNNNNGGNNNNNNGNNGNNNNGNNNNNNNNNNNCNNTNTGGAACGTGCNNNNGNNGNNGNNGNNNNCNANNNN

>seq_tiled_Ecoli_seq_tested_Hinfluenzaee

GGNNANNCNNAGNNTGAAACCCCTGAAGGTCCANACANCGNTTAGATCNNNNNGGNTNTNNNTANNNNNNCACANNNNCCNNNTNNNNNGANNNNNNNGNNANGNNNCNNNNNNGNNGNNCNCTNNCNGGNNNNTNNANAGNNAGGNNGNNNGNGANANNAACANNGCNNNNNNTNCNNGNGNNCNCNNNTNATNNNNNNNANNNNNACCCNNAGNANNNCNNCNNNNNNTNNNANTTGCCGCNNNCNNANNNNANNNNNNNNNNTNNGNNGCNCNNNNNNNNNNNNNNCNNNNNNTNNAGNNNNNNNNNCNNNTNTNATNNNCNNNGGGNNNNNNNNNNNGNNNCNGNANAANNCAGNNNNANGTGCGTTGATGGGTGCGAACANGCAACGTNNACNANNNNNANTNTNNNNNNACNNANNTNNGTCNNNTNNNGNANNNNNTNATNGNNNGNANGGGNNTGCNGGNGNCNGTNGN

>seq_tiled_Ecoli_seq_tested_Mwisconsensis

NGNNNGNGTNCNATCGAAANGNNANAANNNCCANACATCGGNCNGATTANNNNNNNGTCTGNNANCGCACAAACNNNNNNNNNTNGCTTNNNNNNTNNNNGNNNGNNNNNNNNNGNNNNTNNNANNNTAACTGACNNTTANNNNNTCNNNNNNGGGGNTNANNNNNANNNNNNNNNNNNNNGNNNNCNNNNNATNNNNNNNNNCNNCANCCNNGGNNNNNNNNNNNNAGNNNGNNNNNNNNGCNNNNNNNNCNANNNNNNNNNTNTNTNCNGNNCNNNNNNNNNANNNNNNNNNNNTNNNNNNNNNNNNNNNNNTNTGNNNNNNNNNGTGNNGNTNNNGNNNCNTCCNANNNNNNCTGNTNNCCGTGCATTGATNGNTGCGAACANGCAACGTCNNNNTANNNNNNNNNNGNNNNNCGANNNNNNGNNANANNNNNNANNTATGGANCGNGNGNNNNNNNNTGANNCCGGNNNNNNN

>seq_tiled_Ecoli_seq_tested_Pmultocida

NNTGNCNGTCCAATCGAAANGNCTGAAGGTCCANACANTNCGTNANNNCNNNNGGNGTCCGTNTCNGCNNCANTNTNCNNGNNCNNNNNNNCNNCNNANNGANNTNGNGAANTCNGNNGNNTNNNNNANGANNGCGAATNTCNAGNNNGNNTNNNNGCNCNNNNCCGCGTNNTTCNNTNNGNTCAGGCACAANGGNNNNNNANCNCNCNNCCANNNNNGACNNCNNCNTNTGNNATNAGGNGNNTANGNNNCACNTANCNNTTGTTNNGCNNNCNNGNCNNTCGTNNAGNNNNNNNTNNNNGANNNNGNCNNNNTNNNGNCGNTGCGNGGGNNNNGGNNNNTTNTCNGTNCGNCCTNGNGNNANNNGNGATANTCNNNGCAAACATGCAACGTCANGCGNANNTNTNCTNNNNNNNNNNNNTNNNAGNTNNNNAANCCNCAGACANTAGTGCTGTTGNCGNNGNTNNTGGNGTANNT

>seq_tiled_Ecoli_seq_tested_Yersinia_aldovae

CGCGNNNGTCCAATCGAAANGNNNGNNNNNNCANACATCGGNCNGATTAACNNNNNGNNNNNNNNNNNNNCNNNCNACGNTNNNNCNNGGNNNNCTNNGGNNNNNGNNGNNCNGNNNGNNNCCANNNNTNNNGNNNNNNNNAACTNTCTGTCTGCTATTGAAGAANNCANNNTCGNNANCNNTCAGGCGAACTCCAACCTGGATGATGNNCNNAGTNGNNCNNCNNGNNNGNNCANTNGTCGTAGCAAAGGCGANNCNNNNNNNNNNNANNGCGATCAGGTTGNCTATATGGNCNNNNNNGGGNNNNNGNCNTNNNNGNNNGNNNNNGNNNNNTNGGNNCGNCTGGAACACGANGNCGCNNACCGNGCATTGATNGGTGCGAACANGCAACNTCNNNNGNNTNCTANTCTGCGTGNNGANNNNNNGTCGNNNNNNGNCGNTANNGNNNNTGNGNGNNGNNNNNNTGNNNNNNNNNNN

>seq_tiled_Hinfluenzaee_seq_tested_Pmulticoda

NNCGGNGNNGGNCNTGNNGNGGATGAAGGTCCAAACATCGGNTTNNTTNGNGGGNGNGNGGNNTNGTGTNCCNTCGTNNNNNGCCNNNNNNNNNCGNACNCATATCGTAAAGTAGCGNCNCNNNGNANNGNNNCNNNNNNNNNNNNTNNGNNNNANNGNGATGNAGCAAANNNANNCGNCTCANNGNCAAANNCNNCNNNANCNGAANNCTANCNNTTNNNCGGNGNCGNNNTNNNNGGNNGCCGTGAGNGTNGCGCGNTNNCCNNNNGNNNNNGNNCTACANNNNTCNATANGGACGTNTCTCCNNANCGNGCCNNNTNNNGGNCNGNNNAGNCNNNTCCGTTCCTTGAGCCCGACGATGCGAACCGTGCGTTAATGGGTGCAAACATGCAACGTCAAGCGGNACCAACATTACGTGCGGATAAACCATTAGTCGCNGNNNTCNNNNCNNNATCTNCCGCNNNCNNTGNTGTTGNCGCG

>seq_tiled_Hinfluenzaee_seq_tested_E_coli

NGNNGTNGTCNGNNCGAAACNCCTGAAGGTCCGNNCANCGGNCNNNNNNGNGNGNNNNNCGNNNCNNNNNNNNNNNNNNNNGNNNNNNGNNNNNNNGNGGNNNNNNNNNNTNCNGNNGCNNNNNGNNNNGNNNNNNNNNNCNNNNNNNNNNNNNNGNNNNNNNNCNNNNCNNNNNCCNNCNAGNNNNCNNCAGNNNCNNNCNNNNCNNNNGNNNNNNNNGCCGNCGNGGNNGNNGNNGGGGGCNNCNNTNNCNCCNNNGNNNNCNGNNNNNNNNNNNNCNNNNNNNNNNNNNNNNCNNNNCNNNNGNNGNNNCNCNNNNNAGNNGCGGCNNNNNNNNNGNNGNNNGNGNNNGGNNNNNNNCNNNNNNNNGCANTGATNGNTGCGAACANGCAACNNNNNNCCNAGNNGGNNANGNNCGCNGCACANNNGGNNNNNNCCGNGNNGNNCNCNNNNNNGNCCGNNNNNCNNNNNGNNTNNNCN

>seq_tiled_Hinfluenzaee_seq_tested_Egergoviae

NNNNGNNNNNNGNNNGNNNNGGNGGNNNGNNNGNNAGNCNNCCNNNNNGNNNNNNNNNNNNNNNNNNCNNNNNNNGNNNNNNNNNNNNNNNNNNCNNNNGNGNNNNNCNAAGNGGNNGNNNNNGGGNNNGNNNNNNNNNNNNNNNNNNNNNNNNNNNNNNNNGNCGNNNNNNNNNNNNNCNNNNCNNNGNNNNNGGNNNNNNNNNNNNTNNNNCNCNNNNNCNNNNNNNNNNNNNNNNNGGNNGNNNNNNGCNCCCNNNNNNNCNNNNNNNNNNNNNNNNNNNNNNNNNNNNNNNNNNNNNNNNNNNNNNNNNCNNNNNNNNCNNCNNNGNNGNNNNNNNNGNNNGNNNNNGNNNNNGNNNCGNNNNGNGCANNGATNGGTGCGAACANGCAACNNCNNNCNNNNNNGNNNNNNNNNGCNNNANNNNNNNNNNNCNNNGNNNNNGNNNNNNNNANNNCCNNNNNNCNNNNNNACNNCNCN

>seq_tiled_Hinfluenzaee_seq_tested_Erhapontici

NGNNGNNNGGNNNNNNNNGNGGGGGNNGGTCCAAACATCGNNCNNNNNNGCNNCNNTNNNGCNNNNNCNNNNNNNGNNNNNGNNNNCNNNNNNNNNNNGNNGNNNCNNGNNNNNNNNGNTNGNGNNNNNGNNNNNNNNNNNNNNNNNNNNNNNNNNNNNNNNGCNNNNNNNGNCNNCNCCNNGNCNNNGNNNGGNNNNNNNNNNNNNNNNCNNNNCNNNNNCGCNGNNNNCGGNGNNGNGGNNNNNNNNNGNNNNGNNNNNNNCNNNNNTNNNNNNNNNCNNNNNNNNNNNNNNNCNNNNNNNNNNNNNNNNCCNNNNNNGGNNNNNNNGNGGNNGNCNNNNNNNNNNNNNNNNNNNNNNNCNNNNNGNNNNNNGNNNNNNGCANNNNNNCNNCGNCNNNCGNNGNNCNNNNNGNNNGCNGNNNNNNNNNNNNNNNNNGNNNNNNNNNNAAAACCNATCGCNNNNCNNNNNNNNNCNNCC

>seq_tiled_Hinfluenzaee_seq_tested_Hinfluenzae

CGTTTATGTCCGATTGAAANNNCTGAAGGTCCAAACATCGGTTTGNNCAACTCACTTTCNGCTTTTGCACGTACTAATGATTATGGTTTCTTAGAAACACCATATCGTAAAGTGGTTGATGGTCAAGTTNCTGAAGAAATTGAATACTTATCTGTAATTGATGAAGCAAACTACATCATTGCACNGGCAAACTCAAACTTAGNCGAAAATAACCGTTTTACTGATGCTTTCGTNNCNGCTCGTGGTGAANGTGGNGAATCTGGTTTANNTAAACCTGAAGATATTCACTATATGGATGTTTCGNCACAACAAGTGGTNTCNGTTGCGGCNGCNNNNATTCCATTCCTTGAGCNNGACGANGCGANNNGNGCGNNGNNNNNTGCGAACANGCAACGTCAAGCNGTTCCAACATTACGTGCGGATAAACCATTAGTTGGTACAGGNATGGAAAAACCAANCGCACTTGACTCNGGTGNTGCG

>seq_tiled_Hinfluenzaee_seq_tested_Mwisconsensis

NNNGNNNNNGNNNNGNNNNNGGGAGAAGGTCCAAACATCGNCCNCNNNNGNNGGNCNNNCGNNNNNTCNNNNNANGNNNNNGNNNNNNNNNNNNNNNNNNNNNNNNNNNNNNCNNNNGCNNGNNNNNNNGANNGNNNNNNNNNNNGNNNNNNNNCGNNNNNANNNNNNNNNANNATCATNGCACNNCNGCNNGNNNNNNNNNGNNNNNNNNGNNNCNNNGNNGNNGNNNCNNNNNNNGGNNGNGNNNACCGNNNCNNNGNNNNNNGNNNNNNNNNNNNCNCNTNNNNNGNNNNNCCCNCNNCCNNNNNNGNNNCNNNTNTGNCNNNNNTGNNNNNNTNNNNNNNNGNNCNNNNNNGTNNGNGNNNNNGNNCANTGATNGGTGCGAACATGCAACNNNNGNCTNANCCNCNNTNNCNNGCNGNNNANNNNNNNNNNCNNGNNGNCNCNNCTANNNNNNNCGNNNNCNNNNNNNNNNCNNCN

>seq_tiled_Hinfluenzaee_seq_tested_Yersinia_aldovae

GNCNGNNNNGNGNNNNNNGNGGGAGAAGGTCCAAACANCGGCCCNNNNGNNGNNNNNNNCGGCNNGNNNNNCNNNNNNNNNGNNNNNNNNNNNNCNNNNGNNNNNNNNGNNNNNNCNNCNNNNGNNNNNGNNNNNANNNNNNNNNNNNNNNNNNCGNNGNATGAAGCANNNNNNNNNNNNAANACCCNGNNNGNNNNNNNNNNNGNAANNNCNGGNTNNNNCNCNNNGNGTNGNGNNGNNGGNGNNNACNGNNNNGAATCTGGTNNNNNNNNNNNNNNNNNNNNNNGNNNNNNNNNCNNTNNGCNNNCNNNNNCNNCGNNGNNNNNNNNGNNGNCNNNNNGNNGNNGTCNNNCNNNNNNNNNNNNNNNNGCANTGATGGGTGCGAACATGCAACGNCNGNCGNNGNNCCNNNNGNNNGCNCCNAANNCNGCNNNCCNCGNNGNCNNNNCNNNNNNCNCNGCNNNNNNTNNNGGNNNNGCN

>seq_tiled_Kpneumoniae__seq_tested_Erhapontici

CGCGTATGNCNANNCNNANCGNCGGNNNNNNNANACANCGGNCNGNTCNNNGGGNNNNNNNNNNNNNNNCNNNNNNCNGNNNNCNNNNNNNNNCNNCNGNNNNNNNNNGNNNNNNNNGNTNNCNNNNNGGNCNNNNNNNANNNTCGNNNNNNNGNNNNNNNNNNCNNNGGNNNCGNNATCGNTCNGGCGNANANNNNNNNCNNNNNNNNCNGNNNNNGNNNNNNCNTNNNNCNGNCCTGCCGTAGCAAAGGCNNNNCNNNNNCTNNCNNCNGNNNNCNNNTTGACTACATGGACGNTNNNNGNNNGNNNNNNNNTNNNGNCGNTNCGNNNNTGATNNCGNNNCTGNNGNNCNNNGNCGNNNNCCNCGCATTGNNNNNNGCANNNNNGCANCGNCNGGCGGNTNNANNNNNNGNNNNNGNNNNGCCGCTGGTTGGNGNTGNNNNNNNNNNNGNNNNNNNNGNNNNNNNCGGTGTTNNT

>seq_tiled_Kpneumoniae_seq_tested_Ecoli

CGCGTATGTCCANNCNNANCNCCTGAAGNTCCGAACATCGGTCTGANCAACNCCCTGTCCGTGNNCNCNCNNNNNGNCNNANNCGGCTTNNTTGAGNCTCCGNATCGTAAAGTGACCGACGNNGNTNNAGCTGACGAAATTCACTNTCTGTCTGCTATCGAAGAAGGCAACTACNNTATCGNNCNGGCGAACTCCAACCTGGNNNAANNNNNNNNNNNCNNNGNAAANCTGGTNNTCTGNCGTAGCAAAGNCGAATCCAGNNNNTTCNNCNNCGANCNGNTTGACTACATGGACGTATCCANCCAGCAGGNGGTNTCCGTCGGTGCGTNNNTGATNCCGTTNCTGNNNNACGNNGNCGNNNACCGTGCATTGATNNGTGCGAACANGNANCNNCNGNCCNTTNNGNNNCTGNNTCNCGNNNNGCCGCTGGTTGGTNNGGGNNTGGAACGTGNNGNTGNCGNTGANTCCGGTGNANTN

>seq_tiled_Kpneumoniae_seq_tested_Egergoviae

NGNNNCNNNNNNNNNNCNNNNNNGNNNNGTCCGAACATCGGTCTGNNCNACNCCCTGNCCGNGNNNNCNNNNNNNNNNNNNNNCCNNNNNNNGCCNCNGNNNNNTCGTNAAGTGACCGNCGNCNNNNNGNNCCNCGNNNNTCATNNCCNGNNNGNNGNTGNGNNCNNNNNNNNNNGCNNNNNNNCNGCGAACTCCNNCNNNNCCNNNNNNNGCNGNNGNNNNNNCNNTCTGGTTACCTGCCGNAGCAAAGGCNNNNNGGNNNCNNNCANCNGCGNCCNGNTTGACTACATGGACGTATCCNNNNNGNNNNCNNNTNNNGNCGGNGCGNNNNTGATNCCGTNCCTGNNNNACGNNGNCGCNNACCGTGCATTGATGGGTGCGAACANGCANCGNCNGNCGNNNNNGNNNNNNNNNNNCGNNGNNNNGCNGNNNNGNGCCGGTATGGAACGTGCNNNNGNNGNNGNNGNNNCNNNNGNN

>seq_tiled_Kpneumoniae_seq_tested_Hinfluenzaee

GGTNNNNNNNNACNTGAAACCCNNNAAGGTCCANACANNNNNTANNACNNNGNGGNNNNNNNNANNGCNCCANNNANCGAATATGGCTGGNNNNCACNGNNANNNNNCNNTNNNGNGGNNCNCNCTACGGTCCGNNCNNANNNNNGNNNNNNNNGAAANCANCNNNGNNNNNNNNNNNNNNNNNNNNCNNTNATNAANCTNNATGAAANANNCNNNNNNNCNNNNNNNNNTNNNGNNNGNNGCNNNCNNANNNAANNNNNNNNNNTNNGNNGCNCNNNNNNNNNNNNNNCNNNNNNTNNAGNNNNNNNNNCNNNTNTNATNNNCNNNGGGNNNNNNNNNNNGNNNCNGNANAANNCAGNNNNANGTGCGTTGATGGGTGCGAACANGCAACGNGAANNAANNTNANNNNNNNNNNNCNGNNNNNNGTCNNNNNNNNNAGNNNTTNNTNNTNNGNANGGGNNTNNNGGANNNNNNNTC

>seq_tiled_Kpneumoniae_seq_tested_Mwisconsensis

NNNNNNNNNNNANNGNAAACGCCAGAANGTCCANACATCGGTCTGATTAACTNANTGNNNNNNANNNNNNCANNNNNNNNNNNNNNTNNNNNNNCCNNGNNNNNNNNNNNNNNNGNNGNTNNNNNNNANGNNNNNNNTNNNNNNNNNNNNNNNNNGNNTNNNNCNNANNNNNNNANNNNGNNNNNNNNNNNNNTNNNNNNNNNNNNANNNNGCNNNNNNNNNNNNNNNNNNNNNGGNCNNNNCNCNNNNANCNNNNNNNNNNNTNTNTNCNGNNCNNNNNNNNNANNNNNNNNNNNTNNNNNNNNNNNNNNNNNTNTGNNNNNNNNNGTGNNGNTNNNGNNNCNTCCNANNNNNNCTGNTNNCCGTGCATTGATNGNTGCGAACANGCAACNTCAGNCTNCNGNNNTNNNNNNNNNCNNNNNNNNGNNANANNNNGTAGGTATGGANNGNGNGNNNNNNNNTNNNNGNNNNNNNGTN

>seq_tiled_Kpneumoniae_seq_tested_Pmulticoda

GGTNNNNTNCNANTCGAAACGCCTGAAGGTCCANACANNGGNTGNANNNNNNNGGTGTCCGNGTCCNNNNCANAANNCGNNNNCNNCTGGNNNGCNCNGNNANNTNGTGANGTCGNNNNTNNNNNGANGGNNNGCNNNNNNNNANGNTAACNCGNNGNTNANNNNCNCGTNNTTNTNNTCGCTCAGGCGAANTGGNNNNNTNNNNCAACCCNNGNNNNNNCNNCNNNAGCCNNNNAGNGNNGCGNNNNNACCANNNNNCNNTTGTTNNGCNNNCNNGNCNNTCGTNNAGNNNNNNNTNNNNGANNNNGNCNNNNTNNNGNCGNTGCGNGGGNNNNGGNNNNTTNTCNGTNCGNCCTNGNGNNANNNGNGATANTCNNNGCAAACATGCANCGTCAAGCGNTNNNNGTNTNNNNCNNCGANNNGNNANNNANNNNAGTCGGNNNNNNTANTGCTGTTGNCGNNGCNGGTTNNTNNNNN

>seq_tiled_Kpneumoniae_seq_tested_Yersinia_aldovae

CGCGTATGTCNANCCGANACGCCAGAAGGTCCANACATCGGTCTGATTAACTNGTNNNNGNNTATNNNNCCNNNNNCCNNGNNNNNNNGNNCNGAGTCGCCNANCNANGNNCNGNCNGNNGNNNNNNNNGNNGGCTNNNANNNNNGTCNNNNNGCTATTGAAGANNGCANNNTCGTTATCGNTCAGNCGAACNCCAACCTGGNNGANNNCNGNNNNNGGNNNNAAGANCNGNNNGTNNGTCGNNGCAAAGGCGANNCNNNNNNNNNNNANNGCGATCAGGTTGNCTATATGGNCNNNNNNGGGNNNNNGNCNTNNNNGNNNGNNNNNGNNNNNTNGGNNCGNCTGGAACACGANGNCGCNNACCGNGCATTGATNGGTGCGAACANGCAACGTCAGGCGGTTNNTNNTNNNNNTNNCNNNGNNNNGTCNNNNNNNNNCGGTATGGAACNNGNGNGNNGNNNNNNTGGNNNNNNNGTN

>seq_tiled_Mmorganii__seq_tested_Erhapontici

CGCGNATGTNNANNNNCNNNGNNNNNNNNNNNANACANCGGNCTGATCNNCGNNNTGTCTGTTNNNGCNCGNNNNNNNGNTNNNNNCGGGNNNNCNNNNNNANGNNNNNGNNNGCNNNNNGNNNNNNNNGNCGCCGNNANNNNNNNCCNNNCNGNNNNNNNNNCGNNCNNNNNNNNCNNCNNGNCNNNNNTNNNCGNNNNNNNNNNNNNNNNNNNNNNNNNNGCNGNCNNGNNNGCCNGNNNNNGGNNNNNCNCNNNNNGNGNTNNCCGNGGNNGNNNNNNNNGNCNNGTGGACGTTTNCNNNCAGCNGNCGNNNNNCGNNGNNNNGGNNNNGANNNCGNNNCTGNNGNNCNNNGNCGNNNNCCNCGCATTGNNNNNNGCANNNNNGCANCGNCNGGCGGNTNNANNNNNNGNNNNCNNANNTNNGNNNANNNNNGNTGNNNTNGANCGCNCNNCNNNGNNCNNNGNCGGTNTTNCT

>seq_tiled_Mmorganii_seq_tested_Ecoli

CGCGTATGTCNANTCGNAANNNCTGAAGGTCCGAACATCGGTCTGNNCNNNGNNNNNNNNNNTNNCNNNCAGACTAACGAANNCNNCGNGGNNNCNNNGNNNNGNGGNNNTNNGNNNNNNNNNNNNNNGGNCGNCNNNGGNGCCNGNNGNNNNGNTANCGNNNNCCNCNNNGNNGNNNNNNANNCNNNNNNNNGNGNNNNNNNNGNCNNCNNNNNNNNNNGNGNNGNNCNNNCNGNNNGGGNCGNNNNANGCGANNCCNGNNNGNCNCNNNGNTNNCNNNTNNGCCNNGNCNNCGNANNNNGNCNNCNNGCGGTNNNNGNCGNNGCGNNNCTGATCCCGTTNCTGNNNNACGNNGNCGNNNACCGTGCATTGATNNGTGCGAACANGNANCNNCNGNCCNTTNNGNNNNNNNNTNACNNNNNNNNGCNGNNNGNNGNGGGNNTGGANNGNNNNNCNNNNNNTGNNNCCGGNNNNNAN

>seq_tiled_Mmorganii_seq_tested_Egergoviae

NGNNNCNNNNNNNNNNCNNNGGGGGNNGGTCCGAACATCGGTCTGNNCNNNGGNNNNNNNNNNNNCNNNCNGACTAACGNNNNCNCCGGGGNCACNGNGNNNNNNNNNNNNNNGNNNGNNGNNNNGNNNGGNGCCNNNATTCATTACCTGNCNGNNNNNNNNNCCCNCNNNGNNNNNNNCNNGNCNNNGNTANGNNNNNGGNNNNNCNNCNNNGNNNNCNNNNNNGNNNCGNNNGNCNGNNNNNNANNNNNCNNNNNNNNNNNNNNNCGNNNCNNNNNNNNGNGNCNNNNCNNCGNANNNGGNNNGNNNNCNGTTNNNGNCGNNGCGNNNNTGATNCCGTNCCTGNNNNACGNNGNCGCNNACCGTGCATTGATGGGTGCGAACANGCANCGNCNGNCGNNNNNGNNNNNNNNNNNCNNNNNNNGGNNNNNNNNNGNCGGTATGGANCGNNNNNCNNNNNNNNNNGNNNNCNNNNAC

>seq_tiled_Mmorganii_seq_tested_Hinfluenzaee

GGNNNNNTNNNNTNTNCANNNNNNGAAGGTCNANACANNNNNTANNNNNNNNGNNNNNNGNNNNTNNCTNCNNNGNATNNNNNNGNCNGGGNNNCNNNGGNNNGTGGTTNNNNGCNNNNNNNATNNTNTGGCGCCNCNNNNNNNCNNGNNCANGCAATCGAANAAGGCCNANACATCATTGCACAGGCNNNNNNNNCNNNNNNNNNNNGNCNNNNNNNNNCNGNNGNNNNGNNNGNTNGNGNNNNNNANNTCANNGGNNNNNNNNNNNAGGNCTTNNNNTNGNNNCNNGNNCNCNATNNNGNGNNNNNNNCNNNNNTNNNNNNCTCNGNNNNNNNNNNNNNGNNNCNGNANAANNCAGNNNNANGTGCGTTGATGGGTGCGAACANGCAACGNGAANNAANNTNANNNNNNNNNNNCNNNNNNNNGANCNNNCNNGNAGNNNNTANNAGNGTNNNNNANNNNNNTGGANNNNNGNNN

>seq_tiled_Mmorganii_seq_tested_Mwisconsensis

NGNNNNNNNNNNNNNNCNNNGGNNNNNNNNCCANACATCGGTCTGATTAACNNNNNNNNNNNNNNNGCGCAAACTAACGAATATGGTNNGNNNNNNGNGNNNNNNNNANNNNNNCNTGNCGATGNTGNGGTCGNNNNGNNTCATTACCTGNCNGNGNNTGAANCNGGTAACTTCATCATTGCACNGNCGNNTACCGNATNNGANNANGNCNNNNNANNNNNNNNNGNNNNNNNNGNNNGNNNNNNNNNNANCNNNNNNNNNNTNTTCNGCCGNNTANNGNNNNNNNNNNNNNNCGNTNNNNGNNNNCNNNNNNNTGTNNNTGNCNNNNTNGNNNTNNCGNNNCNTCCNANNNNNNCTGNTNNCCGTGCATTGATNGNTGCGAACANGCAACNTCAGNCTNCNGNNNTNTNNNNNNACNNNNNNNNNNNNNNNNNNGNAGGTATNGATCGTGCANNTNCTGTTGANNNCGNNNNNNNN

>seq_tiled_Mmorganii_seq_tested_Pmulticoda

GNNNGTNTNGNANNTNTAAAGGGNNAAGGTCCANACNNNGGNTNNATTTNCGGATTATCCNNTANCNCGNNNNNNTANNCNNNCNNCGGGGNNCCNNNGNNNAGTGNTNNTTNGCGCNGNGNNTATNNNGGCGNNCANGNNNTNAGNGNNCGCNNNATNNCNNCCCNCGGNNNCATCGNNNNNGNNCAGANCATCNCNNTNNNNNNCNNCCNNGNNCGNNNNGTNGAACNGNTNGCCTGCNGCNNAGAANGCNNNNNNNNNNNGANNCGGNNCNTTNNNNTGNNGNGATNCNGCNTTNANNGNANGCCNNCNNNTGNATCNANNNNGNTGNNNANNNTANNTTNTCNGTNCGNCCTNGNGNNANNNGNGATANTCNNNGCAAACATGCANCGTCAAGCGNTNNNNGTNTNNNNNCACNNTGNAAGNNANANNNNNGTCGGNNTNNNNAGTGCGGNNAAGTNTGCNGGTNNCNGGNAT

>seq_tiled_Mmorganii_seq_tested_Yersinia_aldovae

CGCGTATGTCCANNNNNANNGNNNNNNNNNNCANACATCGGTCTGATTAACTCCNNGNCGNGNNNNNNNNCNNNCNNCGNGNCNGNCNGGNNNNCNNNGNNNCNNNNNNNNNNNNNCGNNNGNGNTGTNNNCGCNNCNNNNGCCNGTNNNNCNGNNNNGGNNNCNCNCNNNNANNCNNNCNNNACNCANNNNNGNNCNNNNNNCNNNNGNNNNGNTAGNNNNNCNGNNCNNNNNGNNNGNNNCCNNNNTNTCNNNNNNNGNANNNCNCNNNGCNNNGNNNNNNGNNGTNNNNNNNNNGNNGGNNNNCNNNCNGNTNNNNNCGNNGCNNNNNNNTNNGNNCGNCTGGAACACGANGNCGCNNACCGNGCATTGATNGGTGCGAACANGCAACGTCAGGCGGTTNNTNNTNNNNNNNACNNNCNTNNGNGNANNNNCNNCGGTATGGAACGTGCGGTNGCGGTTGGNGNGNGNNNNGAN

>seq_tiled_Pagglomerans__seq_tested_Erhapontici

NNNNNNNGNNNNNNNNNNNNGGNNGNNNGCNNNNNNANCGGTCNGNTCAACTCCTTGTCTGNTNNCNNNCNGNNNNNTNNNCNNGNNANNNNNCNNCNNNNNGGNCGNNNNNNNNTCGNCGGNCNGNCNGNNNNNCCNNNNNNTNGNNGNNCNNNNNNTGAAGAGGGTAACNACGTNATCGNTCNNNNNCNNANNNGGNNGGNNGNNGNNGNNGNGNNNGNNGNNNNNNNNANGGNNNGNCGNAGCAAAGGCGNNNNNNNCNNNNNCNGNNGNNNNCNNNNTGACTACATGGNCGNNNNNNNNNNGNNCNNNNNNGNNGNCGNNGCGNNNNTGATCCCGNNNCTGNNGNNNGNNNNNNNNNNNCGCGNNNNGNNTNNNNCANNNNNGCANCGNCNCNNGNNNNNANNNNNGCNNGNNGANNNNCCGCTGGTTGGTNCTGNNNNNNNNNGNGNNNNNNNNGNNNNNGGNGNNNNTNNN

>seq_tiled_Pagglomerans_seq_tested_Ecoli

NNCTNNNGTNNNNNCGAAACNNNTGAAGGTNNGAACATCGGTCNGATCAACTCCCNNNCNNNTNNCNNNNNNNNTNNNNNNNNCGNCTNTNGNCCNNNGCNNNNNCGCCNNTGGNNNGNNNNNNTGNTTGCNGNCNCANNNNNCCGNCGGGCNNNNNANGNNNCANCCNNNNNNGGNNTCGNNNNNNNACNNNANGGGGNGNNNNNAGNNGNCGCNNNNNNNGNNNNNNNNCNNGNTNNNCGNAGCAAAGGCGAATCCNNNNCGNNTNGCNGCGNNCNGGTTGACTACATGGNCNNNNNNNNNNNNNNNNNNGNTGTGNNNGGNNCGNNNNTGATCCCGTNNCTGNAACNCGNTGNCGNANNNCNCGNNNTGNTGGGTGCGAACANGNANCNTCANNCCGTTCCGACTCTGCNTGNNGANNNGCCGCTGGTTGGTACGGGTATGGAACGTGNNGNTGCCGNTGNNNCCGGTGTAACN

>seq_tiled_Pagglomerans_seq_tested_Egergoviae

CNCGTCTGCNNNATCGAAACNGNGNNNCGTGNGNACATCGGTCTGNTCAACNNNCNNNNNNNNNNNNNNNCNNNNNNNNNCCNNNNNNGNNNNCCNNCNNNNNNNCGCNNNTNNNTCGNCGNNNNGNCNGNNNGNCCNCNNNGNNGNNGNGNNNNNNNTGAANNGNNCGNNNNNNGCNNNNNNNNNNNNNNNNNNGNGNNNNNNNNNGNNNNNGNNNNNNNNGNNNNNNNNNNTGNCNGCCGNAGCAAAGGCGNNNNGNNCNNNNNCNGCNGCNNCCNGGTTGACTACATGGNNNNNNNGCNNNNNNNNNNNGNNGNNGNCGNNGCGNCNNNGATCCCGTNCCTGGNNNNCGNNGNNGNNGNNNNCGNNNNGNNGGGTGCGAACANGCANCNNCNNNNGNNNNNNNNNNGGNNNNNNGNNGNNNNGNNNNNNNNNNNCNNTNTGGAACGTGCNGNNGNNGNNGNNGNNNNNNNGNNC

>seq_tiled_Pagglomerans_seq_tested_Hinfluenzaee

NNNTNTNTNNNNNNNGNANNANNNGNNNNTNNACNNNNCGNTTAGATCNNANNNGNNNGNTCNNNNNNNNCACANNNNNNACNNNNNTNNNNNNNNNNGCNTTNCCGCCNNNNTNTNGNNNTNGNTTNNGTNGGNNCNCNNNNTNGNNNNNNNNNNNNNNNNNTANNNNNNNNNNNNNNGNNNTNNCNNNNNANANNNNNNGNNGANNNNGGNGNANTTNNTGCTNNTNNNNNGGNCNGNNGNNNNNNNANCNNNNNNNACNNNNTNNGNGGCNNNNNNNNNTNNNGNGNTCNTGTNNCNCTANCGNNNNGGCNTNNNNNTNNCGCNNNGNANNNNNNNNNGNNNNTNAANNNTNNNNNNNNNNNCGNGNNGATGGGTGCGAACATGCAACGTNNACNANNNNNANTNTNNNNNNACNNANNTNNGTCNNNTNNNGNANNNNNTNATNGNNNGTANGGGNNTGCNGGNNNNNANNTN

>seq_tiled_Pagglomerans_seq_tested_Mwisconsensis

NNNTNNNGNNNNANNNNNNNGNNNNNNNNNGNANANANCGGNCTGATTAACTCCTTGNNNNNNGNCNNNNNNNNNNTNNNNNCNNNNNNGNNTNNNCNNANANNNCGCNNNNNNNNNNNTNNNNNNNNNGNCGGNNNNNNNNNNNGNNNNGNNNNNGNNNNNNCNNNNGNNNNNNGNNNGNNNNNNNNNNNNNNNNNGNNNGNTGANNNNGNNNTNNNNNNNNNNNNNNNNNNNGNNNNNNNCNCNAANNNCNNNNNNNNNNNNNNNNGNNGCNNNNNNNNTNNNNATNTNNNNNNNNNCCNNNNNNCNNNNNNNNNNNNNNNNNNNNNNNTNATCCCGTTNNNTCAANACGATGATGCNNNNNGNGNNNNGATGGGTGCGAACANGCAACGTCNNNNTANNNNNNNNNNGNNNNNCGANNNNNNGNNANANNNNNNANNTATGGANCGNGNGNNNGNNNNTGANNCCGGTGNNNNN

>seq_tiled_Pagglomerans_seq_tested_Pmulticoda

NGTGTCTGTCCAATCGAAANGGATGNNNNTGNANNNNNTGCGTNANNTCNNGGNNNNNNNNNNGNTCNNNNACNNNNCNACCNNGNCTNNNATNCNNNNNNANNCCGCNGCGNNATCGNCGGNNNGNCTGTNNGCGCCCNNNGNNGACAAGCNGCNNNNNNCCCNCNNNNANNTNTTATCGCTCAGGNANNCNNNNGANNTGACNNNGNTGNNGNANNNNNNNTNCNNGNTCNNGNNNGNNNCNNNAAAGNCAAANNGCNNNCTNNTTNNNANGNNNNNNNTGNNNNTGTNCGCNNNTTTNTANCTNNNNGNGCNNCTNNCGNGNCGNNGNCANTGGNNNGNNNTCCNTNCNNCANNCNATNACGNGNNNTGNNTNNNGNAAACATGCAACGTCANGCGNANNTNTNCTNNNNNNNNNNNNTNNNAGNTNNNNAANCCNCAGACANTAGTGCTGTTGNCGNNGNTNNTNNNNANNAN

>seq_tiled_Pagglomerans_seq_tested_Yersinia_aldovae

GNCNNNNGNNNNTNNNNANNGNCNNNCNNNGNANNCANCGGNCNGATTAACTCCCNNNCNNNCNNCNNNCNGACCAACGAGNNTGGGNGNNNNGAGACGCCNNNNCGNNANANGNNTGNTCNNNNGNNNNNNGGNNCNNNNNNNNGNNGNGNNNNCNNNNNNCNNNNNNNANTNNTTNTCGNTCAGNNCNNTNNNGNGNNNNNNNNAGNNNNNGNAGNNNNNNCNANNNNNCNTGCTNGTCGNAGCAAAGGCGAATNNNNCNNNNNTNGCCGCGATCAGGTTGACTATATGGNTGTTTCCNCTCANCNCNNNNNNGNGNNNGNNGNNGNNNNNNNGNNNCGGCTGGNNCACGANNNCGNNNNNNNCGNNNNGATGGGTGCGAACANGCAACNTCNNNNGNNTNCTANTCTGCGTGNNGANNNNNNGTCGNNNNNNGNCGNTANNGNNNNTGNGNGNNGNNNNNNTGNNNNNNANNNC

>seq_tiled_PErhaponticiA__seq_tested_Erhapontici

CGCGNATGTCCAATCGAANCGCCGGAAGGTCNANACATCGGTCNGNTCAACNNNTNTNNNNNTNNNNNNCNGANCNNTGANNATGGNNGGNNNCCNGNNNNNNNNNNNNGNNNNNNNNNNNNCGNGNNGANCGNCGAAATTCATTNCCNCNCNGCGNTTGAAGAGGGNNNNNNNNNNNTCGNNCNGNCNAACACCAANCNCNNCNNTNCTNNNNGNNGNNNNNNNNNTNNNNNGNCCTGCCGTAGCAAAGNCNAANNNNNCNNNNNCNGCNNNNNNCNNNNTGACTACATGGACGTTTNCANNCAGCNGNNGGTTNNNGNCNGTGCGNNNNTGATNNCGNNNCTGNNGNNCNNNGNCGNNNNCCNCGCATTGNNNNNNGCANNNNNGCANCGNCNGGCGGNTCNANNTCNGCNNGNNGANNNNCCGCTGGTTGGNGNTGGNNTNNNNNGNGNNGNNNNNNCCNNNNNNNCNNNNGNN

>seq_tiled_PErhaponticiA_seq_tested_Ecoli

CGCNNNNGTCCAATCNNAACNNNNNANNNTCCGAACATCGGTCNGNNCAACTCCCTGNCCNNTGNCNNNNNNNNNGNCNNNNCCNNCNGGGNNNCNGNGNNNNNNNGGGGNNNNNNTNNNGNCNNNNNNNNNGNNNNNCNCNNCNGTNNNNTNGCNNNCGANNNNCNCCTNANNNNCNNNNNNCNGNCNCANNNNNACCNGNNNGNNNCNNNNNGGNGNNGTNNNNNNNNGNNNNTCNGNCGNAGCAAANNCGAATCCNGNNNNTNNNNCNGCGNCCNGGTTGACTACATGGACGNANNNNGNNNNCCNNCGNNNNNNGNCGGTGCGTNNNTGATNCCGTTNCTGNNNNACGNNGNCGNNNACCGTGCATTGATNNGTGCGAACANGNANCNNCNGNCCNNTTNGGCNNNGCNTGNNGANNNGCCGCTGGTTGGTGNGGGNNTGGAACGTGCNGNNGCCNNTGNCTCCGGTGNNGNN

>seq_tiled_PErhaponticiA_seq_tested_Egergoviae

CGNGNCNNCNNNNNCGNNNCNCCGGAAGNTCCGAACATCGGNCNGNTCAACTCCCNNNCCNNNNNNNCNNNNNNNGNNCNNNNNNNCNGGGNCNCNNNNNGNNNNNGNNNNNGNNNNNNNNNCNNGNNGNCCGNCGAAATTCATTACCTGTCTGCTATTGAAGNGGGNGNNNNNNGCNNTNNNNNNCCNNNNTNCNNCNNNNNCGNNGNTNNNNGNNNNGNNGNCGATCTGGTTNCCTGCCGNAGCAAAGNCNNNNCGNNCNNNNNCNGCNGCNNCCNGGTTGACTACATGGNCGNANNNGGNNNGCNGGNGGTTNNNGNCGNNGCGNNNNTGATNCCGTNCCTGNNNNACGNNGNCGCNNACCGTGCATTGATGGGTGCGAACANGCANCGNCAGNCGGNNNNGGNNNGNNCNCNCGNNGNNNNGCNNNNNNNNGNCGGTATGGAACGTGCNGNNNCNGCNNNNGNNNNCNNGGNC

>seq_tiled_PErhaponticiA_seq_tested_Hinfluenzaee

GGNNANNCNNAANNNANCNCNGNNNNNNNNNNANACANCGGTTAGATCNNNGNNNNNNGNNNNGNNNNNNCACAGNNNNTNNTNNNNGNGNNNNCNNNNANNANTNNNNGTCGGNNTNNNNTNNNNNNGNTGGNCCCANNTNNACGGGNNCGGNNGGGNGANNNNNNCNNNAGGNNNNNNNNNNNNNCNNAAAANNNNNCTCNNNANANNNNNNGNNGTTNTGNTGNNNNTNNNGNNNGNNNNNNNCNNANNNNANNANANNNNTTTCGCNGCNNNNNNNNNNNNNGNGNTCNCNNNNNNGNGNNNCCNNNNNTTTNNNNTTTCNNNGGGNNNNNNNNNNNGNNNCNGNANAANNCAGNNNNANGTGCGTTGATGGGTGCGAACANGCAACGNGAANCANCNTNAGTTNGNNCNNNCNNANNNNNGNCNNNTNNNGNAGNNGNTNNTNNNGGNNNGNNGNCTGGNNNNNNNNNNTTN

>seq_tiled_PErhaponticiA_seq_tested_Pmulticoda

NNTGNCNGTCCAATCGAAACGNCTGAAGGTNCAAACNNTGCGTNANNNCTNGGGGNNNNCNNNGTNGNNNNACTNGNCNNTGNNNGNGGGGNNCCCGNGNGNGNTNCGGGTGGGNNNNNNGNNTANNNGNNGNNNNCCNNNGNANGNGNTCTNGCGATTNAACNNNGCGTAANNNNNATCGCTCAGGCNCNNNNNNNNTGTCNNNANNNCNNNGGNAGGNTTGNNNGNNTTTTCNAGNNNNGCNNNNNNACCACNNCNANCTNNTTTNNCNNCNNNNNNNNTNNTNNNGNCNGCNTTNAGGGNANNCNNNNNGTTNNNNNNNNTGCGNGGGNNNNGGNNNNTTNTCNGTNCGNCCTNGNGNNANNNGNGATANTCNNNGCAAACATGCANCGTCAAGCGNNNNNNTANNGNNNNNNCNNNNTNNNANNTANTNNAGTCGGNNTNNNTAGTGNGGNTGCCGNTGACTCTGNTNCNNNT

>seq_tiled_PErhaponticiA_seq_tested_Yersinia_aldovae

CGCGNNNGTCCAATCGAAACGCNANAAGNTCCANACATCGGTCNGATTAACTCCCTGNNNNNNGNNNNNCNGACCAACGAGTATGGTTNNCTNNCNNNNNNTNATCGNNGNNNGNNNNNTNNNGNGGTGACNGNTNCNNANNNNNGNNNNNCTGCTATTGAAGAAGGCNANTTCGTTATCGNTCAGNNNNANNNCNACCNNCNTNNTNNTNNNGGGNGNNGTNGTNNGNNNNNNGTNNGTNGNNGCAAAGGCGAANCANNCNANNNTNGCCGCGATCAGGTTGACTATATGGACGTTGNNGGNNNNCNGNNNNTTGNGNNNNNNNNNGNNNNNTNGGNNCGNCTGGAACACGANGNCGCNNACCGNGCATTGATNGGTGCGAACANGCAACGTCNGGCGGTTNCNACTCTGCGTGNNGANNNNNNGTCNNNNNNNGNCGGTATGGAACGTGNGNNNGNNNCNNGNNNANNNNNGGNN

>seq_tiled_PErhaponticiA_seq_tested_Mwisconsensis

NGNNNGNGTCCAATCGAAACGCCANAAGGTCCANACATCGGNCTGATTAANTNATNNNNNNNNNNCNNNNNNNNNNNNNNANNNNNCNNNNNNNNNNNNNNNNNNNNNNNNNGNNNNNNNNNCNNNNNNNGNNNNNNNCCNCATTACCTGTCTGCGNTTGAAGAAGGNNTNNNNNGNNNGNNNNNNNCNNNNNNCANNNNNNNTNNNNNNNNCNGNNNNNNTGNNNTANNNNNNGNNNNANACNCNANNANCNNNNNNNNNNNNNTNNNCNGCNNNNANNNNNNNNATNTGGNCNNTNNNNNNNNNNNNNNNNTNNNNGNNNNCNNTGTGNNGNTNNNGNNNCNTCCNANNNNNNCTGNTNNCCGTGCATTGATNGNTGCGAACANGCAACNTCAGGCTNNGGANNNTNNNCGNNNCGANNNNNNGNNNNANNNNGTAGGTATGGANNGTGNGNCNGCNGTTGNNTNCGGTGTNNNN

>seq_tiled_Pmultocida_seq_tested_E_coli

NGCNNNGGNNNGGNCNNAACCNNNGNNGGTCCGAACANCGGNCNGNNNNNNGNNCNCNGGNNNNNNNNNCNANNNNNNGNCNNCGNCNGNNNNNCNNAGNNGCGNNNNGCNNGGNNCNGNNNNGNGCCNNGCNCNNCNNNNNCNGNNTNGNCNNNNNNNGNTGNNNNCNNNNNNNGCGCNNNCCNGNCNNNAGNNNNCNNGNNNGCNNNNNNNCNTGNNGNNGNCNNNGCNNNNGNNGGGNGCNNNNANAGNGNCNCNNNNGNNNNTNGNNGNGNNNNNNNNCNNNGNNNNCNCCNNNCNNNNNNNNNNNNNNNCTNNNNNNNGNCGGCNNCNNNGNNNNCGNNTNNNNNNGNNNNNNNNNCNNNNNGNNCNNGGNTCNCNGCGAACATGCAACCGCNNNCNACGNNNNNNNNNNNNNNNNNACNNNNGNNNNNCNNNGNNGGNGNNNNNCANGNNGNNNNNGNNNNNGNCGNCNCNNCN

>seq_tiled_Pmultocida_seq_tested_Egergoviae

NNCNNNGNNNNNNNCNANNCGNNGNNCNNNNNGNNNNNCNNCCNNNGNNGNGNNNNNNNNNNNNNNNCNNNNNNNNNNNNNNNCNNNNNNNNNNNNNNNGGNNGNGNCNNNNNNNNNNNTNNNNGGNCNNNNNCNNCNNNNNCNNNGNNNNCNNNNNNNNNNNCNNNCNNANNNNGNNNNNNNCCNCCNNNNNNNGNNNNNNNNNCNANNNNNNNCNTTNCCGNNGCNGNNNNNGNGGGNNNCNNCNNNNGNNNCNNNNNNGNNNNNNNNNNGGGNNCNNNCNNNNGNNNNCNCNCNNNNNTNGNNNNNNNNNNNNNNNNNNNNNCNGNNNNGNNNNNGGCGNNNGNNNNNNNNNNNNNNNNNNNNGNNGCNNNGNNCCCNGNGAACANGCNNNNNCNGNCNNCGTNGNNNNNNNNNGNNNNANNNNNNNNNNNNNNCGNNGNNNNCNCNNNNNNNNNNNNNNNNNCNGNNGNNGNGNNG

>seq_tiled_Pmultocida_seq_tested_Erhapontici

NNNNNNGNNGNGNNCNAANCGNCGGAAGGTCCAAACATCGGNCNNNNNNGNNCCTTGNCNGNTNCNNNNNNNNNNNNNNGNNGNNNCNNNNNNNNNNNNNNNNNNNNNGNNNGGNNCNNNGNNNNGNCNNNGGNNNNGNNNNNNNNNTNTNCGNCNNNNNNNNCCNNNNNNNNNNGNNNNNGNCNGNNCNNNNNNNNNNNGNNNNNNANNANNNNNNNNNNCGNNGCNNNNNNNNNNGGNNNCNNNNNNAGNGNCNNNNNNGNCGGNNGNNNNNCNNNNNNAGCNNNNNNCCNNCCNNNNNNNGNNNNNNNNNNNNNNNNGNNGGNNGCNNNGNNNNNNNNGNNNNNGNNGCNCNNNNNNNNNNNNNNNNNNNNGCNNNNNGCAAACNNGCAACGTCNGNCNNCGNNNNNNNNNNNNNCNGNANNNNNGNNNNNCGGCGNNGGNNANNNNNNNGCNGCNNNNGNNNNNGNNGNCNNGNNG

>seq_tiled_Pmultocida_seq_tested_Hinfluenzaee

NGNTNNGGNGNGNNANANACCCCTGAAGGTCCAAACATCGGTTTGATCAACTNNCNNNTGNNTNNGNNNCNNGNNNNNNNNNGNGCCNNNNNNNNNNNANCANNTNGCGNNNNNGNNCTNGNNNNNNCCGNNNCNNNNCNNNCNCNNNNNNNCNCAATCNAAGNANNNGNNNNCNNGGNNNGNNNNNCNNANNNNNNNTNNNNTNNNCNNTTACGCANGNNTGNNGNNNNNNNNGGGTNNCGCGNCGNNNGTGNCGNANNNNNNNTTGNNCGNGNNNNANNANNNNGNGATATGGATGTTTCGCCACAACAAGCGGNNNTGNNNGCGGCNNNNNNNNNNNCANTCCTTGTGCATGNCGNTGCNAACCGTGCGTNGATGGGNNCGAACANGCAACGTCAANCANNTCCAACTTTNCGTGCGGATNAANCATTAGTTGNCNNNNNNNNNNGNNNNGCCGNNGNNNTNCNAGTNGNNTCNNNN

>seq_tiled_Pmultocida_seq_tested_Mwisconsensis

CGTGGGNGTNNNNNCNNAACGCCAGAAGGTCCAAACATCGGCCNNNNNNGGNNCCNNNNNNNTNNTNNNNNNNNNNNNNNNNGNNNNNGNNNNNNCNNGNNNNNNNGNNAANNNNNNNNNNNCNNGNCNNNANCNNNNCNNNNNNNTTNNNCGNCNATTGAANNNGNCNNNANNNNNGNGNGANNNCNNNNNNCNNNANNNNNNGNNNNNNNNGGNNNNNNNGNNNNNGNNTNNNNNGNNNNCNNNGNGAGNGNNNNTNNANNNNGNGNNNNNNNNNCNNNNNNNGGNNNNNNNNNCGTTTCCNCNNNNNNNNCNNNTNNGNNNNNNGCGNNNNNNNNNNNNNNNNNTNNNCNNCNNNNGNNNNNNAANNNNNNGNNNNNNNCGAACANGCANNNNNGNNCNNCGNNNNANNNNNGNGNNNCACNNNNCNNNNNNGNNGNNGNCNNNNNACNNNNNNANNNNNTTGNNNNCNNNNNANCG

>seq_tiled_Pmultocida_seq_tested_Pmulticoda

CGTGTCTGTCCAANCGAAACGCCTGAAGGTCCAAACATCGNNTTGNTTNGNGNNNNAGNCNNTNTTGCACGTACCAACGACTACGGTTTCTTANNGNGNGNNCNTNNNAAAGTGGTGAANGGTNAAGTANCACCTNCANNCGCNNNTNNTGNNCCGNGNGAAGAAGGTAAATATGTTATCGCTCAGGCGAACTCGAATCTTGACGAAGAATTACGCTTTACCGATGCGTTTGTTACCTGTCGTGGTGAACACGGTGAGTCTGGTTTATATCGTCCAGATGAAATTCACTATATGGACGTTTCTACACAACAAGTCGTATCTGTGGCGGCAGCGTTAATTCCGTTCCTTNAGCACGACGATGCGAACCGTGCGTTAATGGGTGCAAACATGCAACGTCAAGCGGTACCAACATTNCGTGCGGATAAACCATTAGTCGGTACTGGTATTGAGAAAGCCGTAGCGGTTGACTCTGGTGTAACG

>seq_tiled_Pmultocida_seq_tested_Yersinia_aldovae

NGCNGNGNNNNNNNCNANNCGCCAGAAGGTCCAAACANCGGCCCCNNNNGNGNNNCNCNNGNNNNNNNNNNNGNNNNNNCNGNNNNNNNNNNNNCNNGGGGNNGNNNNNNNNGGNCCNGNNNNNNNNNNGNNNCNNNANNNNNNCNTTTANCGGCNNNNGAAGANGNCNNNANNNNGNNCGNTCAGGCGAAGNNNNNCGNNNNNCCCNNNNNGNANNCNGTNGNNNNNNNNNCNGNNGGGNNCNNCNNGAGNNNCNCNNNNGGNNNTNNNNGNGNNNNCCCNNCNNNCNATATGGACGTTNNCGCNNNNNNNNCNNNNNNNNNGGNNNNNNNGNNNTNNNNNNNNNNNNNNCNCCCTNNCNNCNNNNNNGCNNNGNNNNCNGNGAACANGCNNCNNNNGNCNNCGTNNCNNTNNCGNGCNNNNNNNNNNNNGNNNNNCGNNGNNGNNANANANNCNNNNNCNNGNNGNNCGGNNGNNTNG

>seq_tiled_Pstuartii__seq_tested_Erhapontici

NGCNNANGNNCNNTCGAAANGGGGGNNNNNNNANACANCGGNCNGNTCNNNGGCNTGNCTGTTTNNGCNCGGNTCNNTGNTCNNNNNNNGNNNNCNNNNNNNNNCCNNNCNTNNNGNNCNGNNNNNNNNGNCNNNNNCNNNNNGGNNNGNNCNNNGGNNGANGNNGNCNNNNNCNNNNCCNNGNNNNNGNNNNGNNNNNNNNCNNCNNNNNNCNNNGGNNNNNCNNCNNCCNNGGNNNNNNNNTNNNGTNNNNNNNNNNNNNCNNNCNGNNGCNNNNCNNNNNNNNNNNNNNNCNNNNNNNNGNNGNNNNNNNNNGNNGNCNCNGCGNNNCTGATNNNGNNGNNNNAACNCGNNGNNNNNGNCNGCGCNNTGNNNNNNGCANNANNNCAACGTCNGNCGGNNNNNGNNNNNNNNNNNGNNNNNNNGNNNNCCCNNGNTGNNNTNNNNNNNGNNNNNNNNGNNNNNGNNNNNNAGNNN

>seq_tiled_Pstuartii_seq_tested_Ecoli

NGCNNANGTCCAATCGAAANNNNTGAAGGTCCGAACATCGGTCTGANCNNNGNNNCNNCGGTTTNTGCNCCNNNNNNNGNNNNNNNNNGGNNCCCNNNGNNNGGNCNNNCNNGGGNNNCNNNNNNNNNNGNTGACGAAATTCACTATCNNNCNGCNNNNGANNCCNNNNNNGGNGNNNNCNGNNNNCNNNNANGNNNNNNNNCNNCNANNNNCNNNGGNNNGNNNNNGNNNNNNGNCNNNNNCGNNGNTNNCNNNNNCGNNNNGTTCNGCGGCNACNCGNNNNNNNNNNNNCNCNNNNCNNCGNNNCNNNNNGNNGNGNGNNCCGCGGGNNTGATNNGGNNCCNGGANCNNGNNGNCNNNGNCCGTGCATTGATNGGTGCGAACANGCAACNNNNGNCNNCGTNGGTANCCCCCNNCCNANNGNNGNNNNCNNNNGNGGGTNTGGANNNNNNNGNNGNCNNTGACTCCGGTGTANCN

>seq_tiled_Pstuartii_seq_tested_Egergoviae

NNNNGCNNCNNNNTCGAAACNNNGGNAGGTCCGAACATCGGNCTGNNCNNNGGNNNNNNNNNNNNNNNNNCNNNNNNNNNNNTNNNNNGNNNNNCNNNGNNNNNCNNNNNNNNNNGNNCNNNNNNNNNNNNCNNCNNNNNNNNNNNCCGNNNNNNNNNNGNNNNNNNNNNNGNNNNCTNCNNNNNNNNGNNANGNNNNNNNNNNNCNNNNNNCNGGGGNNNGNCNNNNNNNNNNGNCCNNNNNGGNNGNNNCNCNNNNNNNCNNNNCNGNNGCNNCNNNNNNNNNNTNNNNCNNNNTNNNCNNNGGNNNNNNTNNNNNNNCNNNGCGNNNNNNNNNNGGNNNNNGNNNGNNGNNGNCNNNGNNCGTGCATTGATNGGTGCGAACANGCAACGNCNGGCNNCGNNNNNGNNNNNNGNNGNNGNNNNNNNNNCCNNNGNCGGTANNGNNNNNGCNNCNNNNNNNNNNGNGNNCNNNNNN

>seq_tiled_Pstuartii_seq_tested_Hinfluenzaee

NTNNTTNNTNNNNNTGAAACCCCTGAAGGTCCANACANNNNNTANNNANNNGNNNNNGTNNATTTNNNNNCNNNGCNNGNNNATGGNNGNTNNNCCGAGGTNNGNNNNGNTNNGNGNNNNNNCTNNNNNGNNCGNNCNNAGGNANNCGGNNNGNCGGNCCANNCNNNNGTNNNCNNCNNNGCACAGNNGNCNNNGNNATNNGCANCNNNNNNCNNTGGNNNNNCTTNNNNNNNNGCNNNNNNNNGNGGTNCCNNANNNGNNNNTNTNNGNNGCNNCNNNNCNCCNNNNGNNCNTGTTTCNCTAGNNNNNNNNNNTNNNNNTNNNGCNGGGNNNNNGNNNNGGNNNNNNNNAGNNGNNTNGGACNGTGCGTTGATGGGTGCGAACANGCAACGTCAAGCAGTTNCANNATNNCGTNCNGATAAACCATTAGTTGGTACAGGNNNNAATAGNGNGANNNNNTNTGNTGGNNNCTNGNNN

>seq_tiled_Pstuartii_seq_tested_Mwisconsensis

NNNGNGNGTCCAATCGAAANGNNANAANNNCCANACATCGGTCTGATTAACTCNCNNNNGNNNNTNNNGCNGNCNANNNNATNTNGNNNNNNNNCNNNNNNNNNNNNNTCNGTAGGCGNNGGNTNTNGNGNNNCNACTNANNNNNNNTNNGCGNNGNNTGAANNAGGTAACTTCATCNTNGNANCNNNGNNNNNGNNNNNNNCANCNNTNGNNTNNGGNNNNNTNNNGNNNNNNNNNNGTCGTAACCGTGGTGNGNNNNNNNNNNNNTGNNGCNNNNNNNNTGAATATATGGACGNNNNCCNANNNNAGGTTGTTTCTGTTGCNNNNNNNNNNNNNNNATTCCTTGAACACGATGATGCNAACCGTGCATTGATNGGTGCGAACATGCAACNNNGGNCNNNNNNNNNNTNNNGCNNCGANCNNNNNNNNNCNGGTACAGGTATGGNNNNNGCGGTTGCNNNNGNCTNCGGTGNNNAN

>seq_tiled_Pstuartii_seq_tested_Pmulticoda

CGNGTCTGTNCAATCGAAANGNCTGAAGGTCCANACANNGGNTNGATTANNGNNTNATCCGNNNNCNNGNNNNNNNNNGNNNNNGNNGGNNNNNCCGNNNNANGNCGNTGTANNNGTANNNNNTNTNGNGNNNGCNNNTNNNNAGGNNGNGNNNAGNTTGANCCCNCNGGNANCNNCNCNGCNGNCNNGNNAATNNNTNNNNCGACNANNNNCNNNGGNNNNNATNNNACGGNNGNNCTGNTNGNNCGTGGCCCTGNNCANNCNTTCNGCNNNTANNNNCGTGNGGNANNGCGCNTTTCTNTNGCGNNCNNCTNNGTNNNNNNNNCGNGNGNCTNGGNNCGGCNTGAGTNCGNCNATGCGANCCGNGNGATANTCNNNGCAAACATGCAACGTCAAGCGGTACCNANATCNCNTNAGGACAAACCATTAGNCNNNGNNNNNNTCNAGAGTGCGATTGGGNNNNNTGGTNGNGANCNA

>seq_tiled_Pstuartii_seq_tested_Yersinia_aldovae

NNCNNATGTCCAATCGAAANGNNNGNNNNNNCANACATCGGTCTGATTAACTCCNNGNNGNNNNNCNNNNNNNCCAACGAGNATGGTTNNNNNNCNNNNNNNNGNNNNNCNNNNGNNNNNNCNNNNNGNGNNGNNTCNNNNANNCGNTGNNCTGCTNNGNNNNNNNNCNNNAATNNTNGCNNNNNNNNNNNANGGNNNNCNNCNCCNANNGNCNNNGGGNNNNNNCCNNCNGNNGNNNNCNNCGNNNNNGCCCNNNNCNTNCNNNNTNNNNGNNANNNGNNTGACNACANGGNTGTTTCCNNNNNNNNNNCNTNNGNGGNNNNNGCNNNNTNNNNNNNNNNNCTGGNCCNNGNNNNCNNCGNCCGTGCATTGATNGGTGCGAACATGCAACNTCNGGCGNCNNNNNNNNCTNNNNNCNAANNNNNNNNNNCCCNNNCCGGTATNGAACGTGCGGNNGNGGCNNNNGGNNNNNNNTGN

>seq_tiled_Pvulgaris__seq_tested_Erhapontici

NGCNNANGTCCAATCGAANCGCCNGNNGGTCCAAACATCGGTCNGNTCAACNCCTNGNCTGTTNNNGCACNGNCCNANNNNCGTNNCNGNNNNCCNNNGNNNGNNNNNNNNNCNNNNNNNNNNGCNNNNNNNNNCCNNNNNNGGNTCCNNNNNGNNNNNNNNNCNNNCNNNGNCNNNNCCGNNNNNGNGNNNNNNNCNNCCNNCNCCNNNNNNGNGNNNNCNNCNANNNNNNNNNNCCNNNGCNGNNNNNNCNCNNGNNNNNNNNNNCGCCGNGACCGNNCNNNNNNNNNNNNCGNNTCCGNGNGGNNNNNNNNNGNNGNCGNNNNGNNNCNGNNGGNGNNNNNNNNGCACNNNNNCGNNNNCCNCGCATTGNNNNNNGCANNNNNNCNNCGNCNNNNGNNNNNNGNGNNGNNCNCCNNAGNGGGGNNNNCNNNNGNNNNNNNNNNNNGNNNNGNNNNNNNCGNNNCCGGNGNNNNN

>seq_tiled_Pvulgaris_seq_tested_Ecoli

NGCNCANGTNNNNNCGNANCNCCNGNNGGTCNGAACATCGGTCNGATCAACNNNNNNNNNNNTNNNGNNNCNGNGNNCNNNCGTNCCGGNNNNCCNNNGNANGGNCGNNNNNNNNNNNNANNNGCCNNNNNGNNCNNNNNNNNCTANNNNNCNGCTATCGNNNNCCNCNNNNGNNGNNNCNGNCNGGCNAACTNNNCNNNNNNCNNCNNNNNNNGNNGNNNNNCNNNNNNNNNNNNCCNNNGCNNNNNNNNCNNNNNNGNNNNNNNCCGCNGCNNNNNNNNGGNNCNNGNCCNNNNANNNNNNNGGNNNNNGNNNGNGNNNGNNTGGGNNNGNNNNGNGNNNCNGGNACACGNNGNCGNNNACCGTGCATTGATNNGTGCGAACANGCAACNNNNNCCCNNNNNGGNGNGGNNCCCCNNNGNNNGGNNNNCCNGNNTNGGNNTNNNACGNNNNGNNNCCGNTGNNNCCGGTGNNNCT

>seq_tiled_Pvulgaris_seq_tested_Egergoviae

NNNNNCNNNNNNNNCGNNNCNNNGNNNCNNNNGAACATCGGTCNGATCAACGGNNNNNNNNNNNNCNNNNCNNNNGNCNNNNNNNCCNGNNNCCCNNNGNNNNNCCGNGNNNNNNNNNGNNNNNNNNNNNNNNNCNNNNNNNNNNNCCNNNCNNNNGNNNNNNCNNNCGNNGNNNNNTNCGNNNNNNNGNNNNNNNCNNNNNCNNCCCNNNNNNNNNNNNCNNNNNNNNNNNNNNGNCGNNGCNGNNNNNNCNCNNGGGNNGNNNNNNNNNNCCNNNNNNNGNNNNNNNNCNNNNNNAGGGNNNGGNNNNCNNNNGNNGNCGNNGNGNNNNNNNNNGGGNGNNNGNNNCACGNNGNCGCNNACCGTGCATTGATGGGTGCGAACANGCAACNNNNNTCNNNNNNGNNNNGGNNCNCCNNNNNGNGGNNNCCCNNNNNCGNNNNNNNANGNNNNGNNNNNNNNNNNGNNNNCNNNGNN

>seq_tiled_Pvulgaris_seq_tested_Hinfluenzaee

NNNNNTCNNGNANNAACTNCCNNTGAAGGTCCAAACATCGGTTTGATCNCNGNANNNNNNNNNNTNNNTNTNNNNNNNCNTCGNNTCGGGNNNCCNNNGNNNGGNCTNNANNNGANNGNNNTNGNNNCNNATNTCNNGNNCNNNNNNNGTNNNGCAATCGAANAAGGCCNNNNCNTNNNCNNACANGCNNNNNNGNCGNNNNGNNCCCNNNTNNGNNNNNCNCCTNNNNNNNNNNNTNGNNNCNGANAAATCCCGNCNNNNTGNNTNAGCCGNGNNNANANNNGACNNNNNNNCNNNNNNGNNGNNNNCCCNTNNGNNNNTNNCGNNGGNNNNGNNGNNNGGNNNNCNTNNANNNCNGNNNNANGTGCGTTGATGGGTGCGAACANGCAACGTCAAGCAGTTNGNNAGNNNNNCNNCNNNNNGNAGANANCTNNCNCAGNNNNNNNGCNTGANGNTTNCANTNGNNNAGNNGTTNNT

>seq_tiled_Pvulgaris_seq_tested_Mwisconsensis

CGTGTGNGTCCAATCGAAACGCNNNAAGGTCCAAACATCGGNCTGANTNNNGGGNNGNNNNANNNNNNGNCGNNNNANNCNCNNNANNGGNNNTNNTNGNNANNNNNNNNNNNNNNNNNACTCGCNNNNNNTNNNNGNNNNGNNNNCNNCNNGNNGNNTGAANCNNGTAACTTCATCNTTGNANCNNNGNNNNGNNNNANNNNNNCCNNNNTNNNNNNNNNNNNNNNNNNGNNNNTNCNNANCNNNNNNNNNNCTNNGGNCNNNTACNNNNGNNNNGGNNNNGGNNNNNNNNNCNNNTCCNNANNNCNGNTNNTTNNNGNTGNNNNNNNNTNCNNNNCATTCCTTGAACACGATNCTGNTNNCCGTGCATTGATNGNTGCGAACATGCAACGTCNGGCTGNNNGNNNNTNGGNCNCCNNNNNNNGGNNNNNNNNCNNNGGTATNNNANGNNNNNNNNNTNTTGNNNGNGNNNNNGNT

>seq_tiled_Pvulgaris_seq_tested_Pmulticoda

NGTGNCNGTCCAATCGAAACGCCTGAAGGTCCAAACATCGNGTNANNTNNNNGANNNNNCNNTANCGCGCCCNNANNTNNNNGNNNCGGGNATCCNNNGNNNGGNNANNNCNAGCNNNNTCNCGCCNNCNNNGANGANNNNGNNGNNGCNNNGNCNCTNNCNNCCCNCGGNANCANCNNCGNTGCCCNGNTNNNGNNATTAGCNNNNCNNGNNNGGAGNNTTGANNNNTNTCANNNNCGNCGGNGNCANNGCNCNNNGGTGNNNTNACNNCGTNNNCGANNGCNNNGNNNCCNNATTTCTNNANNANNCCNNTNNGTNNCTGCNNNNGGNNTGATTGGNNGCCNTNANTACGANCTNGNGNNANNNGNGATANTCNNNGCAAACATGCAACGTCAACCGACTNNNGNGNGGNNCCCCNNAGNGNNGGNNANCNNNGCAGGTTTNGNNNGTGNCTNTGCGGTTGACTCTNNNTNNNNT

>seq_tiled_Pvulgaris_seq_tested_Yersinia_aldovae

NNCNCACGTCCAATCGAAACGCCNGANGNTCCAAACATCGGTCNGATTAACNGNNNGNCGNNNGNNNNNNCNNNNGNNGAGTACGGTNGGNNNCCGNCGCCGGNNNNNNANNNCNNNNNACCCGCCCCGNNNNGNNNCNNNNNCTNTCNGNCAGNTANNGNNNNNNNCNNNAATNNTNGNGCTCNGGCNNNNNNNNNTNCGNCNNCCNNNNNNNGNNGNNCNNNNNNNNCGNNNGNNNGGGACNNNNNNNNCNCNNNNNNNNGNTTCNGCCNCCNNNGNANNNGNCNNNNCCNNNNNTNCGNNNNGNNNNNGTNNGNGNNNGNCGNNNNTCTGANTNNNNNNCTGGAACACGANGNCGCNNACCGNGCATTGATNGGTGCGAACATGCAACGNNNGNNGNNNNGNGNNNANNNCCCCNNNGNGGNGNNNCCCNNCGNCNNNNNNANACNTNNGGNNGCGGNTNGNGGANNNNNNNNN

>seq_tiled_RootA__seq_tested_Erhapontici

NGCGTATGTCCAATCGAAANGGGGGAAGGTCCAAACATCGGTCTGNTCANCNNCNNGNNNNNTNNNGCACNGACCNATGNNNNNGNNNNNNNNNCNGNGNNGNNNTNNNNNTNGCGNNNNNGNNGNNNNGNNNCCNNNNNNNNNNNCCNCNCNGCGNNNGAAGANNNCNNNNNNNNCNNCNNNNCNNNNNTNNNCGNNNNNNNNGNCNNNNNNGGNNNNNNNGNNGNGNNNNNNGNNGNNNNNNGNNCNNNCNNNNGCNNNGNNNGNNGNNGCNNNNNGNCNGNCGNCNNNNNNNNNNNNNNGNGGNNNNCNNNNNNNGNCNNNGNGGNGNNNNNGGGNNNNNNNNAACACGATGNNGCNNNNNNNGNNNTGNNNNNNGCANNNNNNCNNCGNCNNNCGNNGNNCCCATNNCNNNNNGNNNNNNNGNCNNCCNNCNCTGGNNNNANNNGNNNNNNNGNNNNNNNNGNNGNCNNNNNN

>seq_tiled_RootA_seq_tested_Ecoli

CGCGTATGTCCAATCGAAANNNCTGAAGGTCCGAACATCGGTCNGATCAACNNNNNNNNNNGNNNNGNNNCNNNNNNNNNNNNNNNNNGNNNNNNNGNGGNNNNNNNNNANNNGCGNGNTGGNNCGNNCGNCNNCNNNNNNNNCNNNNNGNNNGNNNNCGNNNNCNNCNNNGNNGNNNNNNANNCNNNNNNNNGNNNCNNCNNGGNNGCTNNNNGNNNNGNGGNNGCNNNGNNNGTNGNNNNCGNNNNNNNCGNNNNNNCNNNNNNNNNNNGNNANNNGGNNGNCNNNNNNCNNNNNNNNNNGNNNNNNNCNNNNGNNGGCNNNGGGGGGNCNNNGGGGNGCCNGGANCNNNNNGNCNCNNNCNGTGCATTNATNNGTGCGAACANGCAACNNNNNNCCNAGNNGGNNNNNNNNGCNGNATNNNNGGNNNNNNCNNCGGGTATGGANNGGNNNNNNGNCNNTNNNGNNGNCNNNGNN

>seq_tiled_RootA_seq_tested_Egergoviae

NNNNNNNNCCNANNCGAAACNNNGGANNGNNNGAACATCGGTCTGATCANCNNGNNNNNNNNNNNCNNNNCNNNNNNNNNNNNCNNNNNNNNNCCNNNNNNNGNNNNNNGNNNGCGNGNTNNNNGNCNNNNNNCCNNNNTTCATTACCTGNCNGCTNNNGANNCNNNCNNNNNNNNNNCCNNNNCNCNGNTANGNNNNNNNNNCNNNNNNCNNGGNNNNNNGGNNGNNNNGGNNGNNNNNNNNNGNNNNNNCNNNNNNNCNNNNNNNNGNNNCNNNNNGNNNGNCNTNNNNNNNNNNNNNCNNNGGNNNNCNNNNACNNNCNCCGNGGNGNCNNNGGGNNGNNNGNNNGNCCNNGNNGCNNNNNGNGCATTGATGGGTGCGAACANGCAACNNCNNNCNNNNNNGNNNNGGNCCGNNGNAGNNNNNGNNNNCNNNGNCGGTANNGANNGNNNNNNNNNNNNNNNNGNNGNCNAGGNN

>seq_tiled_RootA_seq_tested_Hinfluenzaee

CGTTNTTGTGCGANTGAAACCCCTGAAGGTCCAAACATCGGTNNGATCAACTCACTTTCTGCTGTNNCNNNNNNNGATGATTATGGTTTCTTAGAAACACCATATCGTTNGNCGCNNGNNNNNNANGTTATNNGNNCNNNNNNNNNGGNNCGNGAGNNCCNNNCNNNNNNNNACATCATTGCACAGGCNNNNNNNNCNTTNGATGACNNNNNNGGCNNGNNTGNNGNNNNNNANGNGGNGGNNGTNNNNANCNNNANNGNNNNNNGNNGNGNNGANNNNNNGNGNNNNGNGNATGTTTCGNCACAACAANNNNNTNTCNNNNNNGNNNNNACCNNNGGNNGNANNNNNNNNGNCGNTGCGANCCGTGCGNTGATGGGTGCGAACANGCAACGTCAAGCNGTTCCAACNTTACGTGCGGATAAACCATTAGTTGGTACAGGTATTAANAGNNNGANGNNNNNTTNNGNANNCNTNNCG

>seq_tiled_RootA_seq_tested_Mwisconsensis

CGNGNNNGTCCAATCGAAANGNNAGAAGGTCCAAACATCGGNCTGATTNACNNGNNGGNNNNTNCNNNGNCNNCNNNNNCNNNNNNNNNNNNNNCNNNTCCATNTCNNTNNNCNCNNANNNNNNNGTNGNNCNNNCNGATTCATTACCTGTCTGCGATTGAAGAAGGTAACTTCATCATTGCACNGNCGANTACNGNNNNNNCNGNNNGNNNNNNNNNGNNNNCNGCNNTGGTTNNTTGNNNAGNNNNNANCNCNNNNNNNTTATTCAGCCGTGAACAGGTTGAATATNTGGACGTTTCCNCANNNNNNNCTNTTNNNNNNNNNNNNNNNNNNNTGNCATTCCTTGAACACGATGATGCNAACCGTGCATTGATNGNTGCGAACATGCAACNNNNGNCTNANCCNCNATTACGNGCNGANNANNNNNNNNNCCNNNCAGGTATGGAACGTNCNNNAGCTGTTGNNNNNGNNNNNNTN

>seq_tiled_RootA_seq_tested_Pmultocida

CGTGTCTGTCCAATCGAAANGNCTGAAGGTCCAAACATCGNGTGANTCANNNGGGNTGTCNGTNTCCNNCCNNNGGNNNCNGNNCNCNNNGNNNCGGANCNNNNNNGGGNNGNGCGNNNNCNNTNTNNGGNNNGCNCNNGNNGNNNNGNNNNNGCGANNCACCCNNNCNGNNTCANNGNCCNNGNNNAGAACNTCNCNTNNGGTGANNGTCNNGGTTNTGNTGCCGGNNNGNNCNNNGNGNNCGNNGCNGGCNNNGNNNNGNNNANNCNGGNNGNNGNNTTNGGNNNANNNCGCGTTTCTACAGCNNNANCCCCNNNNNNTGGTGCGNGNTCNNNGGGNNGNCNTGNGTNCNACGATGCGAACCGTGCGNTANTCNNNGCAAACATGCAACGTCAAGCGGNACCAACATTNCGTGCGGANAAACCATTAGTCGNNGCCGNNGTCAATNGTNNNAGGNNGTNTNNNGNTGNNNNTGCN

>seq_tiled_RootA_seq_tested_Yersinia_aldovae

NGCGTATGTCCAATCGAAACGNNNGAAGGTCCAAACATCGGTCNGANTNNNNNNTNNNNNNNNNNNNNNNCCCNCNNCGCNNNCNNNNGNNNNNCNNNNNNNNNGNCNCNNNNGCGCGNTNNNGGNNNNNNNNNCCCNNNNGCCNGTNNANNNGCTANNGANNNNNNCNNNNNNNCNNNCAAGACNCANNNNGGNNCNNNTGATGAAGATNNNNGNNNNNCNGNNNGGNNGNCCGNNNNNGNNNGNACTNNCNNNNTNCNNNNNTNCAGCCGNGANCANNNNNNCTATATGNNNNNNNNCNNNNNTNNNNCNNNNNNNNNNNNCGNGNGNCNCNNNGGNNNNCTGGNCNNANNNNNCNCCANCNGTGCATTGATNGGTGCGAACATGCAACGNCNGNCGNNGNNCCCATTNCGNGCNGATAAACCNNCCNNCCACGNCGGTATNGAACGNNNGTCNGANNNNNGNGNNNNCNGNGNT

>seq_tiled_Senterica__seq_tested_Erhapontici

CGCGTATGTCCANTCGAAACGCCGGAAGGTCNANACATCGGTCNGATCAACNGGTNNNNNNNTNNNNNNCNNNNNNNCGNNCNCNCNNNNNNNCNNCNNNNNCNTNNCGNNNNNNNNGNNNNCGNNNNGGNCGNCGAAATTCATTNCNNCNNNGNNNNNNNNNNCNNNGGNNNCGNNATCGNTCNGGCGNANANNNNNNNCNNNNNNGNNNNCNNNGNNNNNNCCNNNNNNNNGNCCNGCCGNAGCAAAGGCNNNNCNNNNNCTNNCNNCNGNNNNCNNNTTGACTACATGGACGTTTNCNNNCNGCNNNCNNNTNNNGNCGNNNCGNNNNTGATNNCGNNNCTGNNGNNCNNNGNCGNNNNCCNCGCATTGNNNNNNGCANNNNNGCANCGNCNGGCGGNTNNANNNNNNGNNNNNNNNNNNNNGNCGNNNNNNGNTNNNNNNNNNNNNNNNNCNNNNNCNANNTCCGGTGTTNCN

>seq_tiled_Senterica_seq_tested_Ecoli

CGCGTATGTCCANNNNNAACGNTNNANNNTCCGAACATCGGTCNGNNCAACTCCCTGTCCGTGTACGCACAGNCTAACGAATNCGGCTTNNTTGNNCNTNNNNNNNNCNNNCNNNNNNCNNNCGNNNNGGNCGGCGCNNNNCNCNGTNGGNCTGCTATCGAAGAAGGCAACTACNNTATCGNNCNGGCGAACTCCAACCTGGATGAAGNCCNNGGNNNCGCNNNNNNTNNNCCNNNNNGGCGTAGCAAAGNCGAATCCAGNNNNTTCNNCNNCGANCNGNTTGACTACATGGACGNANNNNGNNNGCNGGNNGTNNNNGNCGGTGCGTNNNTGATNCCGTTNCTGNNNNACGNNGNCGNNNACCGTGCATTGATNNGTGCGAACANGNANCNNCNGNCCNTTNNGNNNNNNNNTNACNNNNNNNNGCNGNNTNNNGTGGGNNTGNANCGTGNNGNTANCNNTNNTNCCGNCNNATNN

>seq_tiled_Senterica_seq_tested_Egergoviae

NGNNNCNNNGNNNNCNNNNCNNCGGAAGNTCCGAACATCGGNCTGATCAACTCCCTGTCCGTGTNCGCACAGACTAACGAATNCNGNNGNNNGCCNCNGNNNNGNNNNNNCNNNNNNNNNGNCGNNNNGGNCGNCGAAATTCATTACCTGNCNGCNGNTGNGNNCNNNNNNNNNNGCNNNNNNNCNGCGAACTCCAACCNNNCCNNCNNCCNNGGNNNNNGNNNNNNNNNNNNNNNNNGCCGNAGCAAAGGCNNNNNGGNNNCNNNCANCNGCGNCCNGNTTGACTACATGGNCGNANNNGGNNNGNNNNCNNTTNNNGNCGGNGCGNNNNTGATNCCGTNCCTGNNNNACGNNGNCGCNNACCGTGCATTGATGGGTGCGAACANGCANCGNCNGNCGNNNNNGNNNNNNNNNNNCNNNNNNNNGNNGNNNNNNGNCGGTATGGAACGNGNNNNTNNNNCNNNNGNNNNCNNNGNN

>seq_tiled_Senterica_seq_tested_Hinfluenzaee

GNNNNNTNNNTAGNTANCNCNGNNNNNNNNNNANACANCGNTTAGATCNNNNNGGNTNTNNNTANNNNNNCNCNNNNCGNANNTGGCTGGNNNNNNCNNNNNNGTNNCNNATNNNNTNNNNNNGNNANGGTNNGCCCAANTNNACGGGNNNGGNGAAANCANCNNNGNNNNNNNNNNNNNNNNNNNNCNNTNATNNANNNNNNNNNNNNCCNNNGNGNNNCNACTANCNNTNNNGTNNGNNGCNNNCNNANNNAANNNNNNNNNNTNNGNNGCNCNNNNNNNNNANGNGNTCNCNANNTNGNGNNNNNNNCCNNTGNNNGTNNCNNNGGGNNNNNNNNNNNGNNNCNGNANAANNCAGNNNNANGTGCGTTGATGGGTGCGAACANGCAACGNGAANNAANNTNANNNNNNNNNNACNNNNNNNGGTNNNNNTNNGNANNANNTNATNNNNNGNNNNGNACTCNNGCAGNNNTGNTN

>seq_tiled_Senterica_seq_tested_Mwisconsensis

NNNNNNNNNNNANNCGNAACGCCANAAGGTCCANACATCGGNCNGATTANNNNNNNGTCTGNNANCGCACAAACNNACGAATNNGGTNNNNNNNNCCTNNNANNNNNNNNNNCNNNNNNTNNCGNNNANGNNNGNNCNNCNCATTACCTGTCNGNGNNTNNNNCNNANNNNNNNANNNNGNNNNNNNNNNNNNTNNNNNTGGATGACGANCNNNGNNNNNCNNNNNNNNNNCNNGNNNNNNNCNNNNNNANCNNNNNNNNNNNTNTNTNCNGNNCNNNNNNNNNNNATNTNNNCNNTNNNNGNNNNNNNNNNNNNGNNNNNNNNNNNGTGNNGNTNNNGNNNCNTCCNANNNNNNCTGNTNNCCGTGCATTGATNGNTGCGAACANGCAACNTCAGNCTNCNGNNNTNTNNNNNNACNNNNNNNNGNNNNNNNNNGNAGGTATGGANNNTNNNNNTNNNACNNNNNNNNNCNNNGTN

>seq_tiled_Senterica_seq_tested_Pmulticoda

NNNNGNGTTCCAATCGAAACGNCTGAAGGTNCAAACANTNCGTNANNNCNNNNGGNGTCCGTNTCNGCNNCNNNANNCNNNNNNNGCTGGNNNNNNCTACNNCCTNNCNNNTCNANNNNTNNCGCTNNNGGNGGNCCNCNCGNANGNGNNCNNNNNGNTNANNNNCNCGTNNTTNTNNTCGCTCAGGCGAANTGGNANGNNNNNNNNNNNCCNNNNNNGTCCCCNNNTNTCNTGNNNNTTNNNGNNNNNACCANNNNNCNNTTGTTNNGCNNNCNNGNCNNTCGTNNTGNNNGCNTTNANNGNANNCNNNCNNNTGNNNNTNNNNCGNGGGNNNNGGNNNNTTNTCNGTNCGNCCTNGNGNNANNNGNGATANTCNNNGCAAACATGCANCGTCAAGCGNTNNNNGTNTNNNNNCNCCCNNNNNNGNNCGNGTNNGNNNNNNTNNAANGTGGNGNNNNNACNCCTNTNNNCTNGGNG

>seq_tiled_Senterica_seq_tested_Yersinia_aldovae

CGCGTATGTCCAANCGNNACGNCANAAGNTCCANACATCGGNCNGATTAACNNNNNGNNNNNNNNNNNNNCNNNCNNNCNNNCNNNNNGNNCNGAGACGCCNANNNNNNNNNNNNNNGNNNNCNNNNNNNNNGGCCNNNANNNNNGTNGNNNNGCTATTGAAGANNGCANNNTCGTTATCGNTCAGNCGAACNCCAACCTGGATGATGCCCNNNGNNNNNCNNCNNNNGNNCNNNNNNGNNNNNGCAAAGGCGANNCNNNNNNNNNNNANNGCGATCAGGTTGNCTNTNTGGACGNTNNNGGNNNNCNNNCCNNTGNGGNNGNCNNNGNNNNNTNGGNNCGNCTGGAACACGANGNCGCNNACCGNGCATTGATNGGTGCGAACANGCAACGTCAGGCGGTTNNTNNTNNNNNNCACNNNNNNNNGTNGGTGGGTNNCGGTATGGAACGTGNNNNNNGNACNNNTGNAGNCNNNGTN

>seq_tiled_Smarcescens__seq_tested_Erhapontici

NGNCNNNGNNNNATCGAAACGCCNNNNNGTCCAAACATCGGTCNGNTCAACNGGTNNNNNNNTNNNNNNNNNNNCNNTNNNNNNNGCNNNNNGNNNANNNNNNNNNGNNGNNNNNGTNNNNNCNNGNNGNNNGNCNNNCNNNNNNGNNNNNCNGCGNNNGNNNCNNNNNNNNNNNGCNCNNNNNNNNNNNNNNNNNNNCNNGCNGNTANNNNNGNNNGNNNNNCCNNNANNNNNGNNCNNCGNNGCAAAGGCGAANNNNNCNNGNNCNNCNNNNNNCNNNTTGACTACATGGACGNTNNNNNNNNGCNGNNGGNTGNNGNCNCNGNGGNNNNGNNGNNNNNGNNNNNGNACNNNGNCGNNNNCCNCGCATTGNNNNNNGCANNNNNGCANCNNCNCNNGNNNNNNNNNNGNNNNNNNNNNNNNNNGNCNNNTNNCGNTGNNNTNNNNNGNGNNGNNNNNNNNNNNNNNNCNNNNNNN

>seq_tiled_Smarcescens_seq_tested_Ecoli

NGNNNNNNTNNNGNNNNANCGNNNNANNANNNGAACATCGGTCNGNNCAACTCCCTGTCCGTGTACGCACAGACTAACGAANNCNGCNGGNNNCCCNNGNNNNNNNNNNGNNNNNGTGNNGGCGNGNNGNNNGNNGNAANCNNTGGTCNNNNNGNNNTCGAAGNNGGCAANNANGNNATCNNNCAGNCGAACTCCAACCTGGATGAANANCGNNANNGCGTNGNNNNNNNNNNGGNNGNNNGNNGCAAAGGCGAATCCNNNNNNTTNNNCCNCGACCNGNTTGACTACATGGACGTATCCANCCNNCNNNNNNNNGNNGNNNNNNNGGNGNNNNNNNNGNGGCTGGAACACGNNGNCGNNNACCGTGCATTGATNNGTGCGAACANGNANCNTCANGCNNNNNNNNNNNGGNNNNACNNNNNNNNGNTGNTTNNCGNNGGNNTGGAACGTGCNGNNNCCNNNNGTTGNNCTANNNNN

>seq_tiled_Smarcescens_seq_tested_Egergoviae

GGNNNCNGNNNNNTCGANNCNCCGNNNNNNCNGAACATCGGTCTGNTCAACNCCCTGTCCGTGTNCGCACAGACTAACGNANTCNNCNGGNNCNCNNNNNNNNNNNGNNNNNNNNNNNNNGGCNNGNNGNNCGNCNNNNNNNNNNGCCNGNCTGCTATTGANNNNNNNNNNNNNNNCNNTNNNCNGGCGAACNCCAACCNCNCCNNNANNNNNNGNNGNNNNNNNANNNNNNNNGNNNNCCGNNGCAANGGCGNATNGNNCCNNNNCNGCNGCGNNCNGNTTGACTACATGGACGTATCCNNNCNGCNNNNNNNNGNNGNCNNCNNGNNGNNNNNNNNGNNNCNGNAACACGNNGNCGCNNACCGTGCATTGATGGGTGCGAACANGCANCNNCNCNNGNNNNNGNNNNNNNNCNNCNNNNNNNNGNNNNNNNNCGNCGGTATGGAACGTGNNGNNNNNNNNNNNNNNNNNNNNNNN

>seq_tiled_Smarcescens_seq_tested_Hinfluenzaee

GGCNNNNNNNAAANNNNNNCNGCTNAAGGTCCAAACATCGGTTTGATCNNNNNNGNTNTNNNTANNNNNNCANNNNNNNNANTNNNNNGNNNNNCNNANNNNANTNGGNNNNNNNNTNNTNNNNCNNNNNNGNCNNNNNNGCNNNGNNNNNANNNGGNNGANNNNGGCANNNNGNNNNNGNGNNNNCNNNTNATNNNNNNNANNNAANNNNNTNGNNNNNNNNNTNNNNNNNNGGNGNNNTNNNNNNANNCCNNNNCAANCCTGTTNNGCNGCNCNNNNNNNNNNNNNNNNNNCNNTNNAGNNNNGCTGNNNGTTNGNGNNNNNNGGGGGAGTGNNNNNNNNNNNNCGNANAANNCAGNNNNANGTGCGTTGATGGGTGCGAACANGCAACNNNNNNNNNNNNNANNNGGNNNNNACNNANNNTGNTNCNNNNTNGNNNNNNTTNANANNGGNNANNNNNNNNGTTNNNCNNNNNTN

>seq_tiled_Smarcescens_seq_tested_Mwisconsensis

GNNNNNNGNTNAATCGNAACGCNNNAAGGTCCAAACATCGGNCTGATTANNNNNNNGTCTGNNANCGCANNAANNNNNNNANNNNGCGNNNNNNCNNNNNNNNNTNGNNNNNNNNNNNNNNNCGNGNTGANCNNNNNNNCNNCNNGNNNNNNNGCGNTTGAANNNNCNNNNNNGNNNNNNNGNNCNCNNNNNNTNNNNNNGNNNGANNNNCNCNGCGNNNNNNCNANNNNNNNNGNNNNNNNCNNNANNNNNNNNNNNNNNNNNNTNNNCNGCNCNNNNNNNNNANNNNTNNNNNNTNNANNNNNNNNNNTNGNTTNNGNNNCNNNNNNGNNCNCNNNNNNNCNTNNANNNNNNNCTGNTNNCCGTGCATTGATNGNTGCGAACANGCAACNTCNNNNNNNNTNNNNNNNNNNNNACNNNNNNNNNNNNNNNNNNGNAGGTNTGGAACGTGCAGNNNCTNNTNNTTGNNNTNNNNTN

>seq_tiled_Smarcescens_seq_tested_Pmultocida

GNNNNTTGTCNAATCGAAACGNCTNAAGGTCCAAACATCGNGTGANNNNTNNNGGNGTCCGTNTCCGCNACNCTANNCGNNANCNGNGNGGNNNNAANNANNANNNNNGNTNTGNNTGNTNGNNNNACGNGNGNNACCCNTNNNNGCNNNCTNGCGATTNAANNNNGCANGNTNNNNTNNNCTCAGGNNCNAGGGNNNGNCANCNNTAANCNNGNNNGNNCGNNNNTNNNNCNNGNNCNNCACNNNAAAACCGANTCNNNNNNNTTTNNCCACCNNGNCNNTCGTNNANTNNNNNATANNNNAANNNCNGNNGNNNNTGNTGGNGCNTCACTGATTCCANGGNNNNNNTACGNCCTNGNGNNANNNGNGATANTCNNNGCAAACATGCAACGTCAAGNGNCNNAANNNNGNNNNNACCNNNNTTGNTNGATTNTNGNTNGNNNNANNNGNGNCGTTGCGNTTGACTNTNCTACNNTN

>seq_tiled_Smarcescens_seq_tested_Yersinia_aldovae

GNNNNNNGTNNAANCNNNACGCNNNANGGTCCAAACATCGGTCNGATTAACNNNNNGNNNNNNNNNNNCNNGACCAACGANCNTGGTNNGNNNNCNNAGNNNNGTNGNNNNNNNNNNANTNNNGNNNNGNCNGATGNAATTANCNNTCTGNCTGCTATTGAAGAAGGCANCNTNGTNATCNNTCAGNCGAACTCCAACCTGGATGNTGANNNGNGNGNNTNNGNANNNCTGGTCACTTGTCGTAGCAANGNCGAATCAAGCCNGTTTNNCNGCGATCAGGTTGNCTATATGGNCTNNNNNGGNNNGNCNNNNGNGGNNGTTGGNGCTTCTCTGATTCCATTCCTGGAACNCGANGNCGCNNACCGNGCATTGATNGGTGCGAACANGCAACNNCANNNGNCNNANNNNNNNNNNNACNNNNNNNNGNNGNNNNNCGNCGGTATGGAACGTGCGGNNGCGNNNNGNNGANCNANNGNN

>seq_tiled_Yenterocolitic__seq_tested_Erhapontici

CGCGNATGTCCAATCGAANCGCCNGNNGGTCCAAACATCGGTCTGNTCAACTCNTNNNCNNGTNNNGCNCNGNCCNNTGANNATGGNNGGCNNCCNNNNGNNGNNNNNNNNNNNNNNGNNNNCGNGNNGNNNNNCNCNNNNNNNNNNNNNNNNNNGGNNGNNNCNNNNNNNNNNNGNNNCGGNCNGGCGNANANNNNNCNNNNNNCTNNNNGNNGNAGGNNCNCCNNNNNNNNGGNNNNNNNCNGCAAAGGCGAANNNNNNNNNNNCNNCGGCNNNGCNCNTGACTACATGGACGTTTCNGNNNNGNNNNCNNNTNNNGNNNNNGNNNNNNCNNNGNGNNGGNNNNNGNACNNNGNCGNNNNCCGCGNANNGNNNNNNGCANNNNNGCANCGNCNNCCGGTTCAANNTCNGCNNGNNGNNNTNNNNCNNNNTCNNGCTGGTNTGGAACGNGCNNNNNCGNNNNNNNNNNCCCNGNNN

>seq_tiled_Yenterocolitic_seq_tested_Ecoli

CGCNNNNGTCCAATCNNANCNNCNNCCNNNCCGAACATCGGTCTGATCAACTCCCNGTCCGTGTACGCNCNGNNTNNCNNNCCCNCCNGGNNNNCNGNGGNNGNNNNNGNNNNNNNNNNNNNCNNNNNGNNNGNNNNNNNCCNCTNTCTNNCNGCNNTCGAAGNNGGCAACNACGNNNNNNNCCNGGCGAACNCCAACCNGNNNGNNNNNNGNNGNNGNNNCNCNNNNNNNNNNNNNNNNCGNAGCAAAGGCGAATNCNNCCTGTTNNGCCGNNNCNCGGNTGACTACATGGACNNAGNNGNGNGNNNGNCNNCNNNNGNNTCNGNGGNGNNGNNNNNGNGNCTGGAACACNNNGNCGNNNNNCNTNNANNGNTGGGNGCGAACANGNANCNTCNNNCCNTTNNGNCTCNGCNTGNNGNNNTNNNGCNNNNNCNNGNGGGTNTNGANCGNNCNNCNNCGGNTGNGNNNTCCCCGNNN

>seq_tiled_Yenterocolitic_seq_tested_Egergoviae

CGNGNCNNCNNNNNCNNNNCNNNGNCNNNNNNGAACATCGGTCTGATCAACTCCCNGTCCGTGTNCGCNCNNNNTNNNNNNNNNNNNNGGNNNNCNNNGGNNNNNNNNGNNNGNNNNGNNNNCNNNNNGNNNGNCGNNNNNNNNGNNNNNNCNGCNATTGANNNNNNNNNNNNNNGCTNTNNNNNNGCGAACTCCANCCNNNNANNNANNNNNNNNNGNNNNNNNNNNNNNNNNGNNNNNNGNNGCAAAGGCGNNNNGNNCNNNNNCNNCNNNNNCGNGNNTGACTACATGGNCNNNNNGGTGNGNNNNNCNNCNNNNGNCNCCNNGGNGNNNNNGNGGNGNCNGGAACACGNNGNCGNNNNNCNNNCANNGNTGGGTGCGAACANGCANCGTCNNCCGNNNNNNGNNTGGNCNCGNNNNGNNNNGCCNNNCCNNGNCNGNNNNGNNNNNNCNNCNNNNNNNNNNNNNNCCCCNNNN

>seq_tiled_Yenterocolitic_seq_tested_Hinfluenzaee

GGNNANNCNNAANNTNCNNANNCTNAAGGTCCAAACATCGGTTTGATNNNNGTNGNNNTNNNNANNNCNNNACANNNNNTNNNNNNCNNGNNNNCNNNNNNNNNTNNNNNTNGGNNTNNNNANNNNNCGGNGGCNNCNNANNNNAGNNNNGCGGNGNNNGANNANGGCAANNNGNNNNNNTNNNNNNCNNTAATNNNNNNNNNNNANNNNGGTCGCTTCATTNNTAGTNNNNCGGNNNNGTANNNNNNNNCCNNNNCAAGCCTGTTNNGCCGCNNNNNNNNNCNNNGNGNTCGNNNNNGNGTGTGNANNNCCNCTNGNGTTNTNNGNGNGGNNCNGTNNNNGNNNNCGNANAANNNANNNNNNNNCGCNNTGATGGGTGCGAACANGCAACGTCAAGCANTTNNNNTNNNNNCNNANNNANTNNNNNTNNCTCNNGNNNCANNTANTAGNGNGNCNTNNANNNGNNNGNNCNNNNNN

>seq_tiled_Yenterocolitic_seq_tested_Mwisconsensis

NGNNNGNGTCCAATCGAAACGCCNNAAGGTCCAAACATCGGNCTGNNTNNNNNNNNNNNTNNNNNNNNNNCANNNNNNNNANNNNNCNNNNNNNNNNNNNGNNNNNNNNNNNGNNNNGNTNNNNNNNCGNNCGNTGAAANNNNNNNNNNNNNCGCGGTTGAANNNNCNNNNNNGNNCNNGNNNNNNNNNNNNNNNNNANNNNNNNANNNNGNNTGNNNNNNCNNNNNNNNNNCNNNNNNNNNNNNNANCNNNNNNNNNNNNNNNNTNNNCNGNNNNNNNNNNNNNNNTNTNNNCGTTTCCACACANNNNNCNNNNNNGGNNNCNGCNNTGNNNNNNGNNNNNCTTNNANNNNNNANNNNNNNNNNNNNANTGATGGGTGCGAACANGCAACGTCNGGCTGTTCNNACTNNNCGNNNNNNNNNNNNNCNANNNNNAGCAGGTATGGANCGTGCANNTNTNNNNNCTNNNNCCCNNNNN

>seq_tiled_Yenterocolitic_seq_tested_Pmultocida

NNTGNCNGTCCAATCGAAACGCCTGAAGGTCCAAACATCGNGTGANNTCNNNNGGTGTCCGTGTCCGNNACANTANNCGNNGNNGGCGNGNNNNCNNNGNNNNNNNNTGNNGGNNTNATNTNNNNGANGNNGGATGANNTTCACNNNNNNNTNGCGATTNAANANGNCAANNTNGNCANCGCTCAGGCGAANTGGNNNNNNNGNNNTANTNGTGGNNNTGNCNCNCNNNNNTNNGGGCCNNNCGNNAAAACCGANTCANGNCCNTTTNGCCGCNTNGNNNGNCTNNGTGNCCGNCNCNNTGNNNNNNNGNTNTCTNGNNNNTNNNNGNTANTGNNNNNNNNGNNNCNGTACGNCCTNNNANNTCACNCTNNTGNTNNNGNAAACATGCAACGTCAGNCGNANNTNNNNNGNNNNNNNNTNTTNNCGNNNTCNNNCGGNGNNGNNAANAGNGNCANTNCGGTTGACTCTTNTNCGGNN

>seq_tiled_Yenterocolitic_seq_tested_Yersinia_aldovae

CGCGNNNGTCCAANCGAAACGCCNGNNGNTCCAAACATCGGTCNGATTAACNGNNNNNNTNGCNNNGCACNGACCAACGAGNATGGTTTNCTNNNAACNNNTTATCGTNGNGTGNNCGNTNNNGNGGNGACCGATGNAATTAACTATCTGTCTGCTATTGAAGAAGGCANCNTNGTNATCNNTCAGNCGAACTCCAACCTGCNNACNNNNNNNGNNNGNGACCCCNNNNNGNTCACTTGTCGTAGCAAAGNCGAATCAAGCCNGTTTNGCCGCGATCAGGTNGNNTATATGGACGTTTCCACTCAACNGANNGNGNCCGTTNGNGCTTCTCTGATTCCATTCCTGGAACNCGANGNCNCNNANNNNNNANTGATGGGTGCGAACANGCAACNTCAGNCGGTTCCTACTCTGCGTGNNGATNNGCCGTTGGNGGGTNNCGGTATNGAACGTGCGGNNNCGGTTGACTCAGNGGTAACC

>seq_tiled_YersiniaA__seq_tested_Erhapontici

CGCGNATGTCCAATCGAANCGCCNGNNGGTCCAAACATCGGTCNGNTCAACTCCTTGNCNNNTNNNGNNCNGNCCNNTGANNATGGNNGGCNNCCNNNNGNNGNNNNNGGNNNNNNNGNNNNCNNGNNGNNNNNCNCNNNNNNNNNNNNNNNNNNGGNNGNNNCNNNNNNNNNNNGNNNCGGNCNGGCGNANANNNNNNNNGNNGATGNNCNCNGNNGNNCCNCNANNNNNNNGGNNNNNNGNNGCAAAGNCGAANNNNNCNNNNNCNGCNNCNNNNCNCNNGACTACATGGACGTTNCNGNNNNNNNNNNNNNNGNNGNNNNNGNNNNNNCNNNGNGNNGGCNNNNGNACNNNGNCGNNNNCCNCGCATTGNNNNNNGCANNNNNGCANCGNCNGGCGGNTCNANNTCNGCNNGNNGANNNNNNGCNGNNTNNNGNTGNNNTNNANCGNGCNNNNNCGNNNNNNNNNNCCCNGNNN

>seq_tiled_YersiniaA_seq_tested_Ecoli

CGCNNNNGTCCAATCNNANCNNCNNCCNNNCCGAACATCGGTCNGATCAACTCCCNGNCCNNNNNCGCNCNGNNTNNCNNNNCCNNCNGGNNNNCNGNGGNNGNNNGGGNNNNNCNNNNNNNCNCNNNGNNNGNNNNNNNCCNCTNTCTNNCNGCNNTCGAAGNNGGCAACNACGNNNNNNNCCNGGCGAACTCCAACCTGGANGAANNNNNTNGCNGNNNNNCCNGNGNNNNANNNNNCCGTAGCAAAGGCGAATCCNNNNNNNNNNNCGNCNACNCGGTTGACTACATGGNCNNAGNNGNGNNNCNGNNNGNNNNGNNNTCNGNGGNGNNGNNNNNGNGNCTGGAACACGNNGNCGNNNACCGTGCATTGATNNGTGCGAACANGNANCNNCNGNCCNNTTNGGCNNNGCNTGNNGANNNNNCGCTGGTTNNNGTNGGNNTGGANNGNNCNNCNNCGGNTGNGNNNTCCCCGNNN

>seq_tiled_YersiniaA_seq_tested_Egergoviae

CGNGNCNNCNNNNNCNNNNCNNNGNCNCNNNNGAACATCGGTCNGNTCAACNNCCNGNCCNNNNNNNNNNCNNNTNNNCNNNNNNNCNGGNNNNCNNNGGNNNNNNNNNGNNNNNNNNNNNNCNCNNCGNNNGNCGNNNNNNNNGNNNNNNCNGCNATTGANNNNNNNNNNNNNNGCTNTNNNNNNGCGAACTCCANCCNCNCNGNNNNNCNNNGNNGNNNCNCNNNGGNNCNNGNCNNNNGNNNCAAAGGCGNATNGNNCCNNNNCNGCNGCNNCNNGNNNGACTACATGGNCNNANNGGNGNNGNNNNNNNNNGNNGNCNCCNNGGNGNNNNNGNGGNGNCNGNAACACGNNGNCGCNNACCGTGCATTGATGGGTGCGAACANGCANCGNCAGNCGGNNNNGGNNNGNNCNCNCGNNGNNGNGCNGNNNNNNGNCGGTATGGANNGNNCNNCNNNNNNNNNNNNNNCCCCNNNN

>seq_tiled_YersiniaA_seq_tested_Hinfluenzaee

GGNNANNCNNAANNTNCNNANNCTNAAGGTCCAAACATCGGTTTGATCNNANNNNNNNNNNNNNNNNNNANACANNNNNTNNTNNNNGNGNNNNCNNNNNNNNNNNNGGNTNNGCNTGNNNNCACNNCGGNGGCNNCNNANNNNAGNNNNGCGGNGNNNGANNANGGCAANNNGNNNNNNTNNNNNNCNNTNATNNNNGNNNNNGNNNNNNNNNGNNNNNNNNCCCGGNNNNNCNNGNNGTACNNNNNNNCCNNNNCAANCCTGNTTNGCNGCNNNNNNNNGCNNAGGGNTCGNNNNNNNGTGCAAAAGGTCGNTNGGGNNNTTNGNGNGGNNCNGTNNNNGNNNNCGNANAANNCAGNNNNANGTGCGTTGATGGGTGCGAACANGCAACGNGAANCANCNTNAGTTNGNNCNNNCNNATNGCCGNNNNNNTNNGNAGNNNNTANNAGNGNGNCNTNNANNNGNNNGNNCNNNNNN

>seq_tiled_YersiniaA_seq_tested_Mwisconsensis

NGNNNGNGTCCAATCGAAACGCCNNAAGGTCCAAACATCGGNCTGATTAACTCCNTGTCTGNNNNNGNNNCANNNNNNNNANNNNNCNNNNNNNNNNNNNGNNNNNNNNNNNNNNNNNNNNNCNNNNCGNNCGNTGAAANNNNNNNNNNNNNCGCGGTTGAANNNNCNNNNNNGNNCNNGNNNNNNNNNNNNNTNNNNNNNNNNGNNNNTCNNNGNNNNNNNNCNANANNNNNNNNNNNNNNNGNNANNNNNNNNNNNNNNNNNNTNNNCNGNNNNNNNNNNNGNNNNNTNNNNGTTTCCACACANNNNNNNNNTNNGGNNNCNGCNNNGNNNNNNGNNNNNCNTNNANNNNNNNCTGNTNNCCGTGCATTGATNGNTGCGAACANGCAACNTCAGGCTNNGGANNNTNNNCGNNNCGANNNNCCGCNANTNNNNNTAGGTATNGATCGTGCANNTNTNNNNNCTNNNNCCCNNNNN

>seq_tiled_YersiniaA_seq_tested_Pmultocida

NNTGNCNGTCCAATCGAAACGCCTGAAGGTCCAAACATCGNGTNANNTCNNGGNGNNNNCNNNNCNGTGNCANTANNCNNTGNNNGNGGGNNNNCNNNGNNNNNNNNNGGNNNNNTNNNNCNCNNNNNGNNGGATGANNTTCACNNNNNNNTNGCGATTNAANANGNCAANNTNGNCANCGCTCAGGCGAANTGGNNNGNNNCCGTTNNTCNTGNGNGAGNCCCNANANNCCNNGGNNCNNNCGNNAAAACCGANTCNNNNNNTTNTNNCNNNNNNNNNNGNNTGNGTGNCNNNCNCNNTNNAACGNCNNNNGTGTCCGTNNNNNNGGTANTGNNNNNNNNGNNNNNNTACGNCCTNGNGNNANNNGNGATANTCNNNGCAAACATGCANCGTCAAGCGNNNNNNTANNGNNNNNNCNNNNNNNCNNNNGNNTNNGNNNGNNTCNANAGNGNCANTNCGGTTGACTCTTNTNCGGNN

>seq_tiled_YersiniaA_seq_tested_Yersinia_aldovae

CGCGNNNGTCCAANCGAAACGCCNGNNGGTCCAAACATCGGTCNGATTAACTCCCTGTCTGNCNNNGCACAGACCAACGAGTATGGTTNNCTNNNAACNNNTTATCGNNGNGNGNNCGNTGNNNNGGTGACCGATGNAATTAACTATCTGTCTGCTATTGAAGAAGGCANCNTNGTNATCNNTCAGNCGAACNCCAACCTGGATGATGANNGCCGCNNNTTGGNAGACCTGGTCACTTGTCGNAGCAAAGNCGAATCAAGCCNGNNTNGCCGCGANCAGNTNGANTATATGGACGTTTCCACTCAACAGATNGTGTCCGTTGGNGCTTCTCTGATTCCATTCCTGGAACNCGANGNCGCNNACCGNGCATTGATNGGTGCGAACANGCAACGTCNGGCGGTTNCNACTCTGCGTGNNGANNNNNNGTNGNTGGGTNNCGGTATGGAACGTGCGGNNNCGGTTGACTCAGNGGTAACC

>seq_tiled_Ypestis__seq_tested_Erhapontici

CGCGNATGTCCNANCNAANCGNCGGNAGGTCCAAACATCGNNCNNNNCAACTCCTTGTCTGTTNNCNNNNNNNNCNNTGNTCNNGNNNGGNNNCCNNNNNNNGNNNNNNNNNNNNGNGNNGNCNCNNNGNNNNNCNCNNNNNNNNNNNNNNNNNNGGNNGNNNCNNNNNNNNNNNGNNNCGGNCNGNCGNANANNNNNNNNGNNGATGANCNCNGNGNNNNNNNNNNNNCNCNNNNNNNCNGNNGCAAAGGCGAANNNNNNNNNNNCNTCNNNCNNNNGNNNGACTACATGGNCGNNNNNNNNCNNCNNGNNNCNGGNGNNNCNGCNNNNNNNNCGNNNNNNNNNNNGNNCNNNGNCGCNNNNNNCGNNNGNNNNNNNGNNNNANNNCANCGNCNGGCGGNTCNANNTCNGCNNGNNGNNNTNNNNCNNNNTCNNGCTGGTNTGANNNGNGNNNCNNNGNNNNNNNNNNCCCNGNNN

>seq_tiled_Ypestis_seq_tested_Ecoli

CGCGTATGTCCNNNCNAANCGNCNNCCNNNCNGNNCANCGGNCNGANNNNNGNNCNNNNNNGTNNCNNNNCNNNNNNNGNNCCNNCCGGGNNCCCNNAGGNNGNNNGNNNNNNNNNCNCNCNCNCNNNGNNNGNNNNNNNCCNCTNTCTNNCNGCNATCGAAGNNGGCAACNACGNNNNNNNCCNGGCGAACTCCAACCTGGANGAANNNCNTNGNGGNNNNCCNNNNGNTCNNNNNGNCCGTAGCAAAGGCGAATCCNACCTGTTNNNCNGNCNCACGGNTGACTACATGGACGNNNCCNNNCNGCNNCCNNCNGNGGGCTNNGNGGNGNNNNNGNNNNNNCNNNAACACGNNGNCGCNNACCGTGCANTGATNNNNGCGAANANGNANCNNCNGNCCNNTTNGGCNNNGCNTGNNGNNNTNNNGCNNNNNCNNGNGGGTNTGGANNGNGNNCNNGCGGNNGNNNCNTCCCCGNNN

>seq_tiled_Ypestis_seq_tested_Egergoviae

CGNNNCNNNNNGNNCNNTNCGNNGNCNCNNNNNNNNNNCNCNCNNNNCNNNGNNCNNNNNNNNNNCNNNNCNNNNNNNNNNNNNNNNNGGNNNNCNNNNGNNNNNCGNNNNNNNNNNNNNNNCNCNNCGNNNGNCGNNNNNNNNGNNNNNNCNGCNNTTGANNNNNNNNNNNNNNGCTNTNNNNNGNCGAACTCCANCCNCNCNGNNGNNCNNNGNGGNNNNNCNNNNNCGNNGNNNNNNNGNNGCAAAGGCGNANNGNNNNNNNNCNNCNNCCNCNNGNNTGACTACATGGACGNNNNNNNNNNNNNNANCNNNNNNGNCCCCNNNGNGNNNNNGNNNNNNNCNNAACACGANGNCGCNNNCCGNGNANNGNNNNNTGCGNNNNNGCANCGNCAGGCGGNNNNGGNNNGNNCNCGNNNNGNNNNGCCNNNCCNNGNCNGTNTGGANNGNGNNNCNNCNNNNNNNNNNNCCCCNNNN

>seq_tiled_Ypestis_seq_tested_Hinfluenzaee

GGNGNNTCTNNGATTGAACCNCCTGAAGGTCCAAACATCGGTTTGATCAACNNNNNNNNNNANANNNNCNNANNGCNTGNNNATGGNNNGNNNCCCNANGNANNTCGCCGTNANNNNGNNNNCNCNNCGGNGGCNNCNNANNNNAGNNNNGCGGNGNNNGANNANGGCAANNNGNNNNNNTNNNNNNCNNTNATNNNNGNNNNNGNNGNNNGNNGCNNNTNNGNNNANNNNNCNNTNCNGTNCNNNCNNNCCNNNNCAAGCCTGTTNNNCCGCNNNNNANNNNGGGGNNNNCNCNCNNNANNNNGNCNNNCCNNTNNGTNGNCCNGNGNGNNNCNNTNNNNGNNNANNNANAANNCANNNNNNNNNTNNNTNATGGGTGCGAACANGCAACGNGAANCANCNTNAGTTNGNNCNNANNNANTNNNNNTNNCTCNNGNNNCANNTNATAGNGNNNNGGCGNTNNNNTCNNNCNNNNNN

>seq_tiled_Ypestis_seq_tested_Mwisconsensis

NGNNNNNNNNGGNNCGNAACGCCAGAAGGTCCAAACATCGNCCNNNNNNNNNCCTTGNNNNNTNNNGCNCNGNNNNNNNNANNTGNNNGGNNNCNNNNNNNNGNNNNNNNNGNNNNNNNNNNCNNNNCGNNCGNTGAAANNNNNNNNNNNNNCGCGGTTGAANNNNCNNNNNNGNNCNNGNNNNNNNNNNNNNTNNNNNNNNNNGNNNCTCNNNGNGNNNNNGAAGANNNNCCNGNNNNNNNCNNNANCNNNNNNNNNNNNNNNNNCNNCNGNNNNNNNNNNNNNNNNNNNNNNNNNNNGNAGNNNCNANNNNNNNNGNNNNCNNNNNNGNANNNNNNNNNNCNTNNNANNNNNNCTNNNNNNNGNNNANNGATNNNTGCGAACANNCAACNTCAGGCTNNGGANNNTNNNCGNNNNNNNNNNNNNCNANNNNNAGCAGGTATGGAACGTGNNNNNGNNNNNNNTNNNNCCCNNNNN

>seq_tiled_Ypestis_seq_tested_Pmulticoda

NNTGGNGTCNNAATCGAAACGCCTGAAGGTCCAAACATCGGNTNNNNTNNAGNNNNNNNGTANNTNNNGCCNNNANTNGNGNNNGNNGGGNNNCCCNANANNCNGGNCGNNTANNNCANCNCCGNNNNGNNGGATGANNTTCACNNNNNNNTNGCGATTNAANANGNCAANNTNGNCANCGCTCAGGCGAANTNGNNNGNNNCCGTNCNCCNNNGCNTTTNTNCTNTCGNNCCNGNNCNGTNCGNNAAAACCGANTCANGNCCNTTTNGCCGCGTNCNNANNCGTCNNGNCNNNNNTNNNNCNNGNCNNTNNNCGGNNGNNNNCGNGGTANTGNNNCCCNNTNNNCANTACGNCCTNNNNNNTNTNTNNNNNNTTNNNNNANNAANGCANCGTCAAGCGNNNNNNTANNGNNNNNNNNTNTTNNCGNNNTCNNNCGGNGNCGTNANNNGNGAGNCTNCGNNNNGNTCTTNTNCGGNN

>seq_tiled_Ypestis_seq_tested_Yersinia_aldovae

CGCGTATGTCCAANCGAAACGCCAGAAGGTCCAAACANCGGCCCNNNTNNCNNNCTGTNNNNCNNNNNCNNNNCCAACGAGTATGGTTNGCNNCCANCNCCTNNTNNNNNNNNGNCCGNTGGNGNGGTGACCGATGNAATTAACTATCTGTCTGCTATTGAAGAAGGCANCNTNGTNATCNNTCAGNCGAACNCCAACCTGGATGATGANNGCCGCNNNNCNNCNNCNGNNCNCNNNTGTCGTAGCAAAGGCGAATCAAGCCNGTTTNGCCGCNANNNGNNNNNANNTATNNNNNCTANNNNNCNGCNNNNNGTGTCCGTTGGTGCTTCTCTGATTCCATTCCNGGAACACGANGNCGCNNANNGNGNANTGANNNNTGCGAACANGCAACGTCNGNCGGTTNCNACTCTGCGTGNNGATNNGCCGTTGGNGGGTNNCGGTATNGANNGNGCGNNNNCGNNTGNCTCAGNGGTAACC
