# Supplementary material for: Reconstructed Ancestral Sequences Improve Pathogen Identification Using Resequencing DNA Microarrays
Source: PLoS One. 2010 Dec 20;5(12):e15243. doi: 10.1371/journal.pone.0015243 (PMC3004854; doi:10.1371/journal.pone.0015243)
Supplement: Table S1 — Call rate and accuracy values obtained after hybridization of seven strains on each of 18 sequences tiled on the PathogenID resequencing microarray. The divergence corresponds to the uncorrected nucleotide sequence divergence between the rpoB sequence of tested strains and tiled sequences. (DOC) [file pone.0015243.s003.doc]

**Table S1.** Call rate and accuracy values obtained after hybridization of seven strains on each of 18 sequences tiled on the PathogenID resequencing microarray. The divergence corresponds to the uncorrected nucleotide sequence divergence between the *rpoB* sequence of tested strains and tiled sequences.

|  | Cfreundii | **CitSalEscEntA** | Eaerogenes | Ecoli | Hinfluenzae | Kpneumoniae | Mmorganii | Pagglomerans | **PErwiniaA** | Pmultocida | Pstuartii | Pvulgaris | **RootA** | Senterica | Smarcescens | Yenterocolitica | **YersiniaA** | Ypestis |
| --- | --- | --- | --- | --- | --- | --- | --- | --- | --- | --- | --- | --- | --- | --- | --- | --- | --- | --- |
| ***Y. aldovae*** |  |  |  |  |  |  |  |  |  |  |  |  |  |  |  |  |  |  |
| % of divergence | 14.17 | 11.17 | 14.17 | 13.17 | 29.2 | 13.57 | 16.17 | 16.17 | 8.98 | 27 | 16.37 | 20.16 | 18.36 | 13.77 | 9.38 | 3.79 | 1.79 | 5.79 |
| Accuracy | 79.35 | 85.2 | 75.97 | 83.59 | 43.4 | 80.66 | 69.55 | 78.02 | 87.5 | 51.9 | 71.1 | 66.39 | 68.91 | 82.33 | 90.64 | 96.49 | 99.75 | 95.03 |
| Call rate | 57.9 | 58.1 | 54.1 | 53.7 | 35.4 | 57.4 | 46.1 | 48.6 | 62.1 | 37.9 | 45.7 | 49.9 | 49.9 | 55.8 | 62.7 | 77.6 | 82.2 | 71.7 |
| ***E. rhapontici*** |  |  |  |  |  |  |  |  |  |  |  |  |  |  |  |  |  |  |
| % of divergence | 11.57 | 10.77 | 11.37 | 13.37 | 30.6 | 12.57 | 13.97 | 14.97 | 7.18 | 27.8 | 19.16 | 18.56 | 18.96 | 10.97 | 12.57 | 14.57 | 12.17 | 14.57 |
| Accuracy | 87.99 | 87.05 | 86.77 | 78.54 | 44 | 86.32 | 76.09 | 82.99 | 93.25 | 52.4 | 64.78 | 72.53 | 70.39 | 87.1 | 80.13 | 79.73 | 81.52 | 78.31 |
| Call rate | 43.6 | 47.0 | 42.8 | 40.0 | 20.8 | 39.8 | 38.6 | 40.7 | 52.8 | 29.6 | 33.3 | 38.2 | 37.5 | 45.5 | 34.8 | 45.5 | 44.2 | 39.6 |
| ***E. gergoviae*** |  |  |  |  |  |  |  |  |  |  |  |  |  |  |  |  |  |  |
| % of divergence | 7.98 | 4.79 | 7.58 | 8.38 | 31.3 | 8.98 | 14.97 | 15.57 | 7.58 | 30 | 19.96 | 21 | 20.36 | 9.98 | 9.78 | 14.57 | 13.37 | 15.77 |
| Accuracy | 90.79 | 95.69 | 90.69 | 89.88 | 43.7 | 89.76 | 75 | 79.9 | 90.12 | 31.2 | 67.35 | 64.94 | 69.19 | 87.85 | 91.31 | 75.9 | 76.89 | 70.93 |
| Call rate | 59.1 | 63.1 | 58.5 | 51.8 | 23.9 | 51.2 | 41.1 | 40.7 | 55.1 | 23.3 | 30.8 | 32.3 | 36.1 | 53.5 | 48.2 | 40.9 | 44.4 | 36.1 |
| ***M. wisconsensis*** | |  |  |  |  |  |  |  |  |  |  |  |  |  |  |  |  |  |
| % of divergence | 17.96 | 17.76 | 17.36 | 18.36 | 25 | 15.37 | 16.57 | 20.95 | 12.37 | 27.4 | 17.56 | 10.18 | 19.36 | 19.16 | 17.96 | 16.77 | 19.16 | 17.96 |
| Accuracy | 63.38 | 69.3 | 66.53 | 64.85 | 48.9 | 59.1 | 74.09 | 37.82 | 63.22 | 52.9 | 76.79 | 66.81 | 82.2 | 64.79 | 64.98 | 70.21 | 70.19 | 58.38 |
| Call rate | 39.6 | 42.8 | 43.8 | 37.3 | 31.7 | 46.8 | 46.8 | 19.9 | 31.4 | 30.8 | 42.6 | 53.7 | 59.7 | 39.6 | 36.3 | 36.5 | 40.7 | 33.8 |
| ***E. coli*** |  |  |  |  |  |  |  |  |  |  |  |  |  |  |  |  |  |  |
| % of divergence | 3.79 | 5.19 | 4.99 | 0 | 29.7 | 4.59 | 14.17 | 13.17 | 9.38 | 28.7 | 18.16 | 18.36 | 18.96 | 7.58 | 11.17 | 13.37 | 12.37 | 14.57 |
| Accuracy | 91.11 | 87.87 | 89.47 | 94.27 | 51.6 | 91.49 | 70.09 | 77.48 | 77.15 | 50.3 | 64.23 | 62.12 | 62.67 | 82.83 | 78.43 | 75.18 | 75.17 | 68.55 |
| Call rate | 79.9 | 65.8 | 70.6 | 82.8 | 34 | 73.8 | 46.1 | 56.4 | 53.7 | 35.8 | 49.5 | 42.6 | 40.5 | 63.3 | 56.2 | 53.2 | 55.8 | 54.3 |
| ***P. multocida*** |  |  |  |  |  |  |  |  |  |  |  |  |  |  |  |  |  |  |
| % of divergence | 27.97 | 28.17 | 28.57 | 28.97 | 18.1 | 29.17 | 29.36 | 28.57 | 27.77 | 0 | 26.39 | 26.78 | 24.21 | 29.96 | 29.36 | 26.98 | 27.38 | 27.38 |
| Accuracy | 46.3 | 50.53 | 47.27 | 43.71 | 62.5 | 48.74 | 39.52 | 45.29 | 45.14 | 87.47 | 49.19 | 49.35 | 56.17 | 43.02 | 47.64 | 43.9 | 47.87 | 41.38 |
| Call rate | 50.5 | 53.7 | 51.6 | 53.9 | 64.2 | 52.0 | 54.9 | 51.8 | 54.3 | 91.4 | 58.5 | 58.1 | 62.1 | 50.9 | 56.0 | 57.4 | 53.0 | 54.7 |
| ***H. influenzae*** |  |  |  |  |  |  |  |  |  |  |  |  |  |  |  |  |  |  |
| % of divergence | 28.97 | 29.36 | 28.77 | 29.36 | 0 | 29.36 | 28.17 | 31.15 | 29.36 | 19.1 | 25 | 26.98 | 15.87 | 29.96 | 31.35 | 28.17 | 28.97 | 27.77 |
| Accuracy | 44.71 | 44.3 | 44.25 | 45.26 | 94.79 | 47.3 | 47.3 | 41.18 | 38.39 | 74.09 | 56.77 | 45.14 | 73.7 | 41.18 | 42.31 | 40.33 | 39.27 | 44.45 |
| Call rate | 37.9 | 42.1 | 41.7 | 42.8 | 90.8 | 40.7 | 40.9 | 37.1 | 41.3 | 53.7 | 50.1 | 48.2 | 62.9 | 40.7 | 37.9 | 45.3 | 46.1 | 47.2 |
